# A systematic analysis of nucleosome core particle and nucleosome-nucleosome stacking structure

**DOI:** 10.1038/s41598-018-19875-0

**Published:** 2018-01-24

**Authors:** Nikolay Korolev, Alexander P. Lyubartsev, Lars Nordenskiöld

**Affiliations:** 1 School of Biological Sciences, Nanyang Technological University, 60 Nanyang Drive, Singapore, 637551 Singapore; 2 Department of Materials and Environmental Chemistry, Stockholm University, 10691 Stockholm, Sweden

## Abstract

Chromatin condensation is driven by the energetically favourable interaction between nucleosome core particles (NCPs). The close NCP-NCP contact, stacking, is a primary structural element of all condensed states of chromatin *in vitro* and *in vivo*. However, the molecular structure of stacked nucleosomes as well as the nature of the interactions involved in its formation have not yet been systematically studied. Here we undertake an investigation of both the structural and physico-chemical features of NCP structure and the NCP-NCP stacking. We introduce an “NCP-centred” set of parameters (NCP-NCP distance, shift, rise, tilt, and others) that allows numerical characterisation of the mutual positions of the NCPs in the stacking and in any other structures formed by the NCP. NCP stacking in more than 140 published NCP crystal structures were analysed. In addition, coarse grained (CG) MD simulations modelling NCP condensation was carried out. The CG model takes into account details of the nucleosome structure and adequately describes the long range electrostatic forces as well as excluded volume effects acting in chromatin. The CG simulations showed good agreement with experimental data and revealed the importance of the H2A and H4 N-terminal tail bridging and screening as well as tail-tail correlations in the stacked nucleosomes.

## Introduction

About 85% of DNA in chromatin exists in the form of regular protein-DNA complexes called nucleosomes^1^. In most eukaryotes, chromatin at the first level of its organization is a linear array of uniform structural units, nucleosomes, formed by 150–210 base pair (bp) of double stranded (ds) DNA and an octamer of highly conserved histone proteins. The most regular central part of the nucleosome is the nucleosome core particle (NCP), which consists of 145–147 bp DNA wrapped as about 1.8 turn left-handed super helix (SH) around the wedge-like octamer histone core formed from the two H2A/H2B histone dimers and the (H3/H4)_2_ tetramer^2–4^. Positively charged Lys^+^ and Arg^+^ amino acids (a.a.) of the “histone-fold” domain form a distinct charged region which directs the DNA wrapping on the lateral surface of the histone octamer (HO)^2,3,5–7^.

The NCP is a polyanion-polycation complex with a net charge of about −148 e, comprising a negatively charged central particle (−236 e, with −294 e from DNA and +58 e from the globular part of the HO, gHO). Eight flexible and highly basic N-terminal tails from each of the histones and two C-termini of the H2A histone, with a total charge +96 e, are attached to the gHO and protrude out from the core domain of the NCP (Supplementary Table S1). The tails facilitate interactions between neighbouring nucleosomes^8–18^. Observations *in vitro* show that array folding and inter-array aggregation occurs as a result of electrostatic interactions due to increased monovalent salt or addition of Mg^2+^ or other multivalent cations, with NCP aggregation displaying a similar behaviour^8,10,13,19,20^. The positively charged histone tails interact not only with the negatively charged DNA of its own NCP but also with neighbouring nucleosomes, with the linker DNA, and with other non-histone nuclear proteins^6,21,22^. These functions of the histone tails may be modified by covalent post-translational modifications of its amino acids.

Linker DNA of a variable length (10–70 bp) connects the NCPs to form a linear array. *In vitro*, the nucleosome arrays fold yielding a fibre of approximately 30 nm diameter whose detailed structure is actively studied and still a matter of debate^23–29^. The relevance of the 30-nm fibre *in vivo* has also recently been the subject of discussion, some authors suggesting an irregular “melted” polymer phase as the major form of chromatin *in vivo*^30–32^ (and references cited in^32^). However, the *in vivo* existence of the 30-nm fibre is supported by recent work^33^.

However, it is clear that a principal element of condensed chromatin is the close stacking contact between the surfaces of the wedge-shaped cylindrical NCPs. The NCP-NCP stacking has been experimentally observed in NCP crystals^3,4,34–36^, in NCP liquid crystalline phases^37^, in the crystals of the 197 bp chromatosome^38^ and tetranucleosome^24^, in folded nucleosome arrays^23,25,39^, and in cryo-electron microscopy (cryo-EM) images of frozen isolated native chromatin^40,41^. Single molecule measurements indeed demonstrate that the nucleosome-nucleosome stacking is energetically favourable^29,42–45^.

20 years after the first atomic resolution structure^3^, a large number of NCP structures are reported (now about 150 entries). Comparison between these structures reveals that binding of the DNA on the histone core is flexible, but has certain structural restrictions. The majority of all 120 protein-DNA contacts occur between charged phosphate groups of DNA and Lys^+^/Arg^+^ of the histone core^46–48^ and this illustrates that electrostatic interactions dictates the NCP formation.

However, a systematic analysis of the major structural elements in these, in particular the nucleosome stacking has not been undertaken. Furthermore, no convention for parameters that define these major structural elements has been suggested. The development of a convention for the description of NCP-NCP contacts, similar to the generally accepted scheme for the characterisation of double stranded DNA and RNA^49^, is therefore timely. Here, we introduce an “NCP-centred” coordinate system to describe the relative three-dimensional positions of atoms and molecules in the NCP system (including other NCPs). This proposed coordinate frame gives a general representation of the NCP as a flat cylinder and the identification of these coordinates does not require calculation of the superhelical path of the nucleosomal DNA. We analyse over 140 crystal structures containing NCPs, with respect to the DNA superhelical parameters as well as NCP-NCP contacts. Furthermore, combining the information extracted from the analysis of NCP-NCP contacts in the crystals with results of our GC Langevin molecular dynamics (MD) computer simulations of NCP self-association, we reveal and discuss the electrostatic forces responsible for nucleosome interactions. Our analysis demonstrates that the histone N-terminal tails H4 and H2A play key roles in stabilization of the NCP stacking and that tail-tail coordination defines the preferences for the NCP orientation in the NCPs stacks.

## Results and Discussion

### Overview of NCP structures

The Supplementary Table S2 presents basic information for pdb entries of crystal structures that include NCP. The vast majority of the 147 entries listed in Supplementary Table S2 have been obtained for a single NCP; formed by 145–147 bp of DNA and having a P2_1_2_1_2_1_ crystal packing. Following the first crystallization of the NCP^34^, the breakthrough report of the NCP structure at atomic resolution^3^, led to the determination of NCPs with histones from various organisms, histone variants as well as complexes of the NCP with small ligands. Important contributions included the tetranucleosome^24^, a complex of the NCP with the peptide LANA (latency-associated nuclear antigen)^50^ and the chromatosome^51^. A number of protein – NCP crystal structures has been obtained and were recently reviewed^52^. The progress in cryo-EM microscopy has resulted in determination of the atomic or nearly atomic resolution structures of single NCP^53–55^, NCP with linker histones^38^ and nucleosome arrays^39^.

### Coordinate system for description of the NCP structure and NCP-NCP contacts

**Figure 1:**
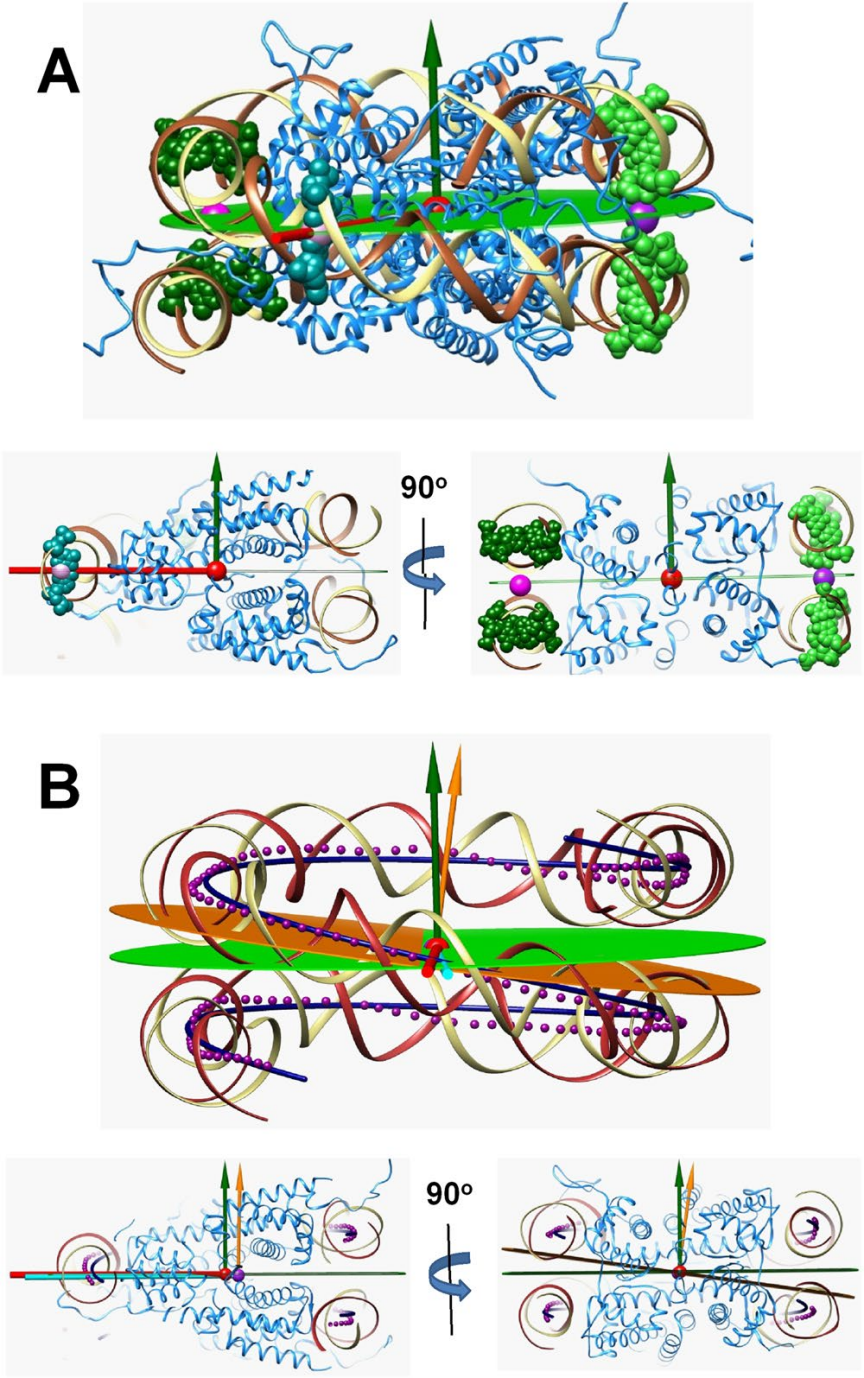
(**A**) Definition of the NCP-centred coordinate system. The top cartoon shows how the atomic 1KX5 structure was used to define COM (red sphere), symmetry axis (red stick), NCP plane (green plane) with the green vector showing the normal to the NCP plane. Lower left and right cartoon are slices through the NCP structure explaining the details of the definition of the coordinate system. Atoms of the DNA used to create coordinates are shown as spheres: coloured cyan is the central “0” b.p that defines symmetry axis; atoms in light and dark green are used to build the NCP plane. (**B**) Comparison of the suggested NCP-centred coordinate system and the coordinate system based on the DNA symmetry approximated as superhelix (SH). The top cartoon presents a general view, lower left and right drawings illustrate details. The Curves+ program^90^ was used to build the sequence of the DNA double helix shown as magenta points at the level of each DNA bp. The dark blue line shows the ideal superhelix (SH) structure fitted to the 129 central points of the dsDNA axis. The magenta sphere shows the centre of the SH; the yellow-orange plane and the vector are respectively the SH axis and plane drawn thought the SH centre normal to the SH axis; the light cyan stick is the dyad axis of the SH. NCP symmetry axes and plane as in (**A**).

Figure 1A shows the scheme used to define the principal parameters in an “NCP-centred” coordinate system, placed at the centre of mass (COM) of the globular part of the histone octamer (gHO; shown as a red sphere in Fig. 1A). The symmetry axis is drawn as a line connecting the gHO COM and the COM of one (for the NCPs with 145 and 147 bp DNAs) or two (for the crystals with 146 bp DNA) DNA base pairs in the middle of the DNA chain (red rod in Fig. 1A). The NCP plane (green surface in Fig. 1A) was defined using coordinates of the DNA base pairs situated in the two opposite DNA chains about 90° from the symmetry axis (these DNA atoms are highlighted in Fig. 1A by light- and dark-green spheres). First, two vectors normal to the planes formed by the symmetry axis and each of the COMs of the base pairs were drawn (shown in Fig. 1A as light- and dark-green spheres). Next, the NCP plane was defined by setting a median of these two vectors as a normal vector to this plane.

**Figure 2:**
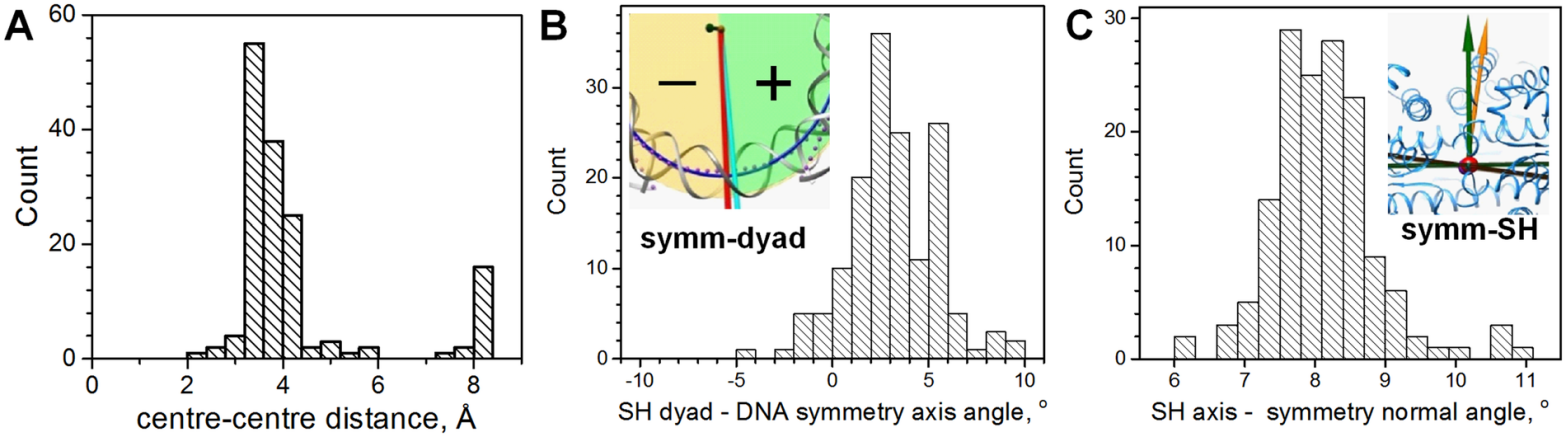
Statistics of mutual orientation of the DNA superhelix (SH) and the NCP symmetry axes in the published NCP structures. (**A**) Distance between centre of the DNA SH and centre of the suggested NCP-symmetry coordinate system. (**B**) Angle between SH dyad axis and the NCP symmetry axis. Inset in the graph shows the positions of the symmetry axis (red line marked “symm”) and SH dyad axis (cyan line marked “dyad”) observed in the 1KX5 crystal^4^). The green area is for positive values of the angle, orange one is for negative angles. (**C**) Angle between the SH axis and the normal to the NCP plane. the inset in the graph illustrates a typical case (1KX5 NCP^4^) of positions of the normal-to-plane vector (green arrow marked “symm”) and the SH axis (orange arrow marked “SH”).

In structural studies of the NCP, the symmetry of the particle is commonly based on a superhelical symmetry of the DNA with specific diameter, pitch and dyad axis that divides the superhelix into two symmetrical halves^2,7,34,35,56–58^. Figure 1B compares the two coordinate systems: based on the NCP symmetry and built as described above (Fig. 1A); and using a least square fitting to an ideal SH using the coordinates of the 129 central dsDNA axis points. For most of the NCP structures, the distance between the origins of the two systems is close (in the range 3–4 Å; Fig. 2A). The centre of the SH (magenta sphere in Fig. 1B) is positioned almost on the symmetry plane of the NCP (green surface in Fig. 1B).

Interestingly, for most NCP structures the origin of the coordinates for the 145–147 bp DNA double helix (using coordinates of the DNA axis) coincides very well with the NCP COM of the globular part of the HO introduced in this work (within less than 2 Å; see Supplementary Fig. S1). E.g. in the illustrative case of the 1KX5 structure, the distance between the centres is only 0.7 Å. The observation that the COM of the gHO coincides with the centre of the DNA superhelix is in fact not trivial and somewhat unexpected. Indeed, the atomic coordinates used to define these reference points are different and the only connection one can find between the two sets of atoms is that the DNA positioning on the HO surface is quite strictly directed by the location of the positively charged arginines^2,3^.

The angle between the dyad and the symmetry axes in the two coordinate systems is small and in the range 0–6° for most NCPs (shown respectively as cyan and red rods in Fig. 2B). The major difference between the coordinates suggested in this work and the frame based on the DNA SH is in the orientation of the NCP plane (or equivalently in the orientation of the SH axis and normal vector of the symmetry plane). The angle between these axes is in the range 6–10° for the published NCPs (Fig. 2C, green and orange vectors and planes). If the SH direction is used to build the NCP plane, then this plane looks skewed and cuts through the DNA helices, clearly misrepresenting the orientation of the NCP cylinder (Fig. 1B). Consequently, we consider the present symmetry-based coordinate system to be a better representation of the NCP geometry since it gives a correct orientation of the NCP cylinder.

The NCP-centred coordinate system can be used to characterise the position and orientation of any atom, molecule or structure relative to the NCP. Supplementary Figure S2 shows this set of parameters (distance to the centre, rise, shift, shift orientation). In this work we concentrate on description of NCP-NCP contacts that requires an extended set of parameters given below. However, we will first analyse parameters of the DNA superhelix in the published NCP structures.

### DNA structure in the NCP crystals

**Figure 3:**
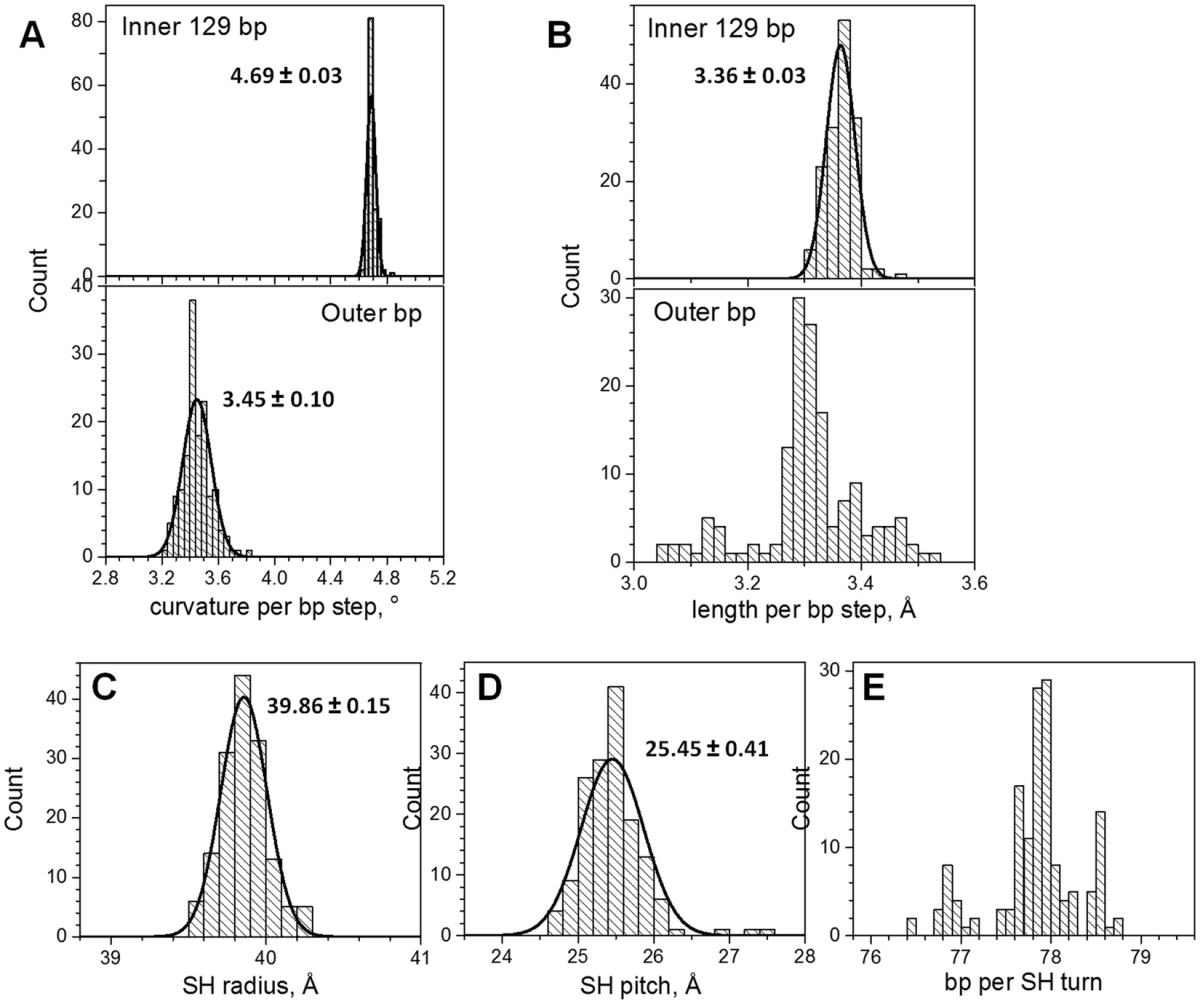
Results of analysis of published NCP crystal structures. (**A**) DNA curvature per bp step calculated for the central 129 bp (top) and outer dsDNA (bottom). (**B**) DNA length per bp step calculated for the inner 129 bp (top) and for the outer dsDNA (bottom). The superhelical radius (**C**) and pitch (**D**). (**E**) Number of DNA base pairs per one turn of the DNA superhelix in the NCP crystal structures. Curves show the best fit to a normal distribution; numbers near the peaks are respective mean value and standard deviation of this distribution.

Figure 3 presents statistics of parameters of the DNA superhelix in the published NCP structures. Supplementary Table S3 gives the detailed data. Bending of the DNA in the inner 129 bp compared to the short DNA stretches at the NCP entry/exit is clearly different (Fig. 3A). The inner DNA has more than one degree per bp larger curvature than the 8–9 bp DNA sections at the ends. It has previously been shown that bending/kinking of the DNA is inhomogeneous and dependent on the DNA sequence^56–58^. However, the highly conserved structure of the HO forces the inner 129 bp DNA loop to conform to restrictions posed by the precise positioning of the key HO residues, which results in the overall bending of the inner 129 bp being strictly fixed.

Furthermore, the inner 129 bp nucleosomal DNA is on the average stretched compared to the double helix of the outer DNA (Fig. 3B). The radius (Fig. 3C) and the pitch (Fig. 3D) of the DNA superhelix calculated from the coordinates of the axis of the inner 129 bp DNA is well-fitted by a normal distribution. The SH radius varies within less than 1 Å (between 39.5 and 40.5 Å) while the SH pitch shows a broader distribution (variation is about 2 Å, between 24.5 and 26.5 Å). The number of base pairs accommodating one full turn of the DNA superhelix in the NCP varies in the range 76.5–79 bp (Fig. 3E). This reflects the ability of the DNA sequence to stretch or contract to accommodate restrictions posed by the DNA-binding sites of the HO.

### Parameters describing the NCP-NCP contact

**Figure 4:**
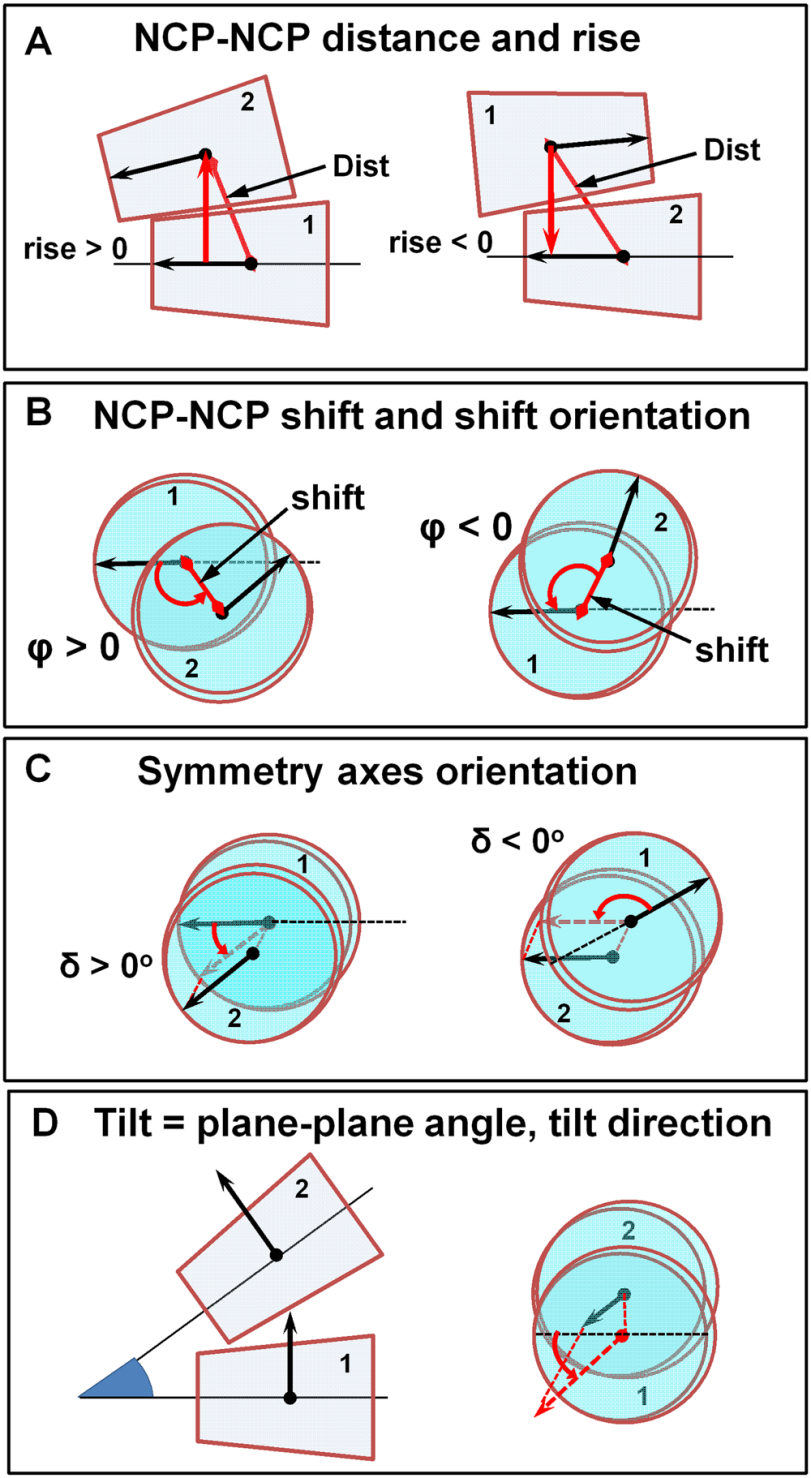
The set of parameters defining the mutual NCP-NCP position and orientation in the NCP-centred coordinate system. (**A**) Distance and rise between NCP1 and NCP2. (**B**) The NCP1-NCP2 shift is characterized by the distance from the COM of NCP1 to the projection of the COM of NCP2 on the NCP1 plane. The angle φ, between the NCP1 symmetry axis and the line connecting the projection point to the NCP1 COM is also defined. (**C**) The mutual orientation of the NCP1 and NCP2 symmetry axes is defined by the angle (δ) between the symmetry axis of the NCP1 and the projection of the symmetry axis of NCP2 on the NCP1 plane. (**D**) The angle between the NCP1 and NCP2 symmetry planes or between the normal vectors to these planes.

To describe the mutual orientation of the two NCPs in the NCP-NCP contact one needs an extended set of parameters compared to the simpler situation when the position of a single point is defined in the NCP-centred coordinate system (Supplementary Figure S2). Furthermore, the set of parameters can be applied for characterisation of multi-NCP structures that can also include other components (e.g. proteins). The NCP-NCP contact may be characterised by the following parameters (Fig. 4):

1. Distance between COMs of the NCP1 and NCP2 (“Dist” in Fig. 4A);
2. Rise, the distance from the COM of the NCP2 to the NCP1 plane. The rise may be positive or negative depending if the NCP2 is above or beneath the NCP1 (Fig. 4A). Since in this work only pairwise NCP-NCP contacts are analysed, the absolute value of rise is used.
3. Shift of the NCP2 relative to the NCP1 measured as the distance between the COM of NCP1 and the projection of the COM of NCP2 on the NCP1 plane (Fig. 4B);
4. Orientation of the shift defined as the angle (*φ*) between the NCP1 symmetry axis and the line connecting the shift point with the COM of the NCP1. The angle *φ* varies in the range −180° and +180° being positive or negative depending on counter clockwise or clockwise turn relative to the symmetry axis (Fig. 4B);
5. The angle (δ) between the symmetry axis of the NCP1 and the projection of the symmetry axis of the NCP2 on the NCP1 plane (Fig. 4C). The term symmetry axes *orientation* is used instead of “angle”. The angle δ varies in the range −180° and +180°;
6. The NCP-NCP tilt is the angle between the planes of the NCP1 and NCP2, which is equivalent to the angle between the normal vectors to the NCPs planes (Fig. 4D, left).
7. The NCP-NCP tilt direction is the direction in which the plane of the NCP2 is tilted relative to the symmetry axis of NCP1 and is defined as the angle between the symmetry axis of the NCP1 and the projection of the plane-normal vector of the NCP2 on the NCP1 plane (Fig. 4D, right).

In principle, six parameters are sufficient to fully define the mutual positions of two bodies (such as NCPs). However it may be practical to use NCP-NCP distance instead of the combination of shift and rise.

### Parameters of NCP-NCP stacking in crystal structures

**Figure 5:**
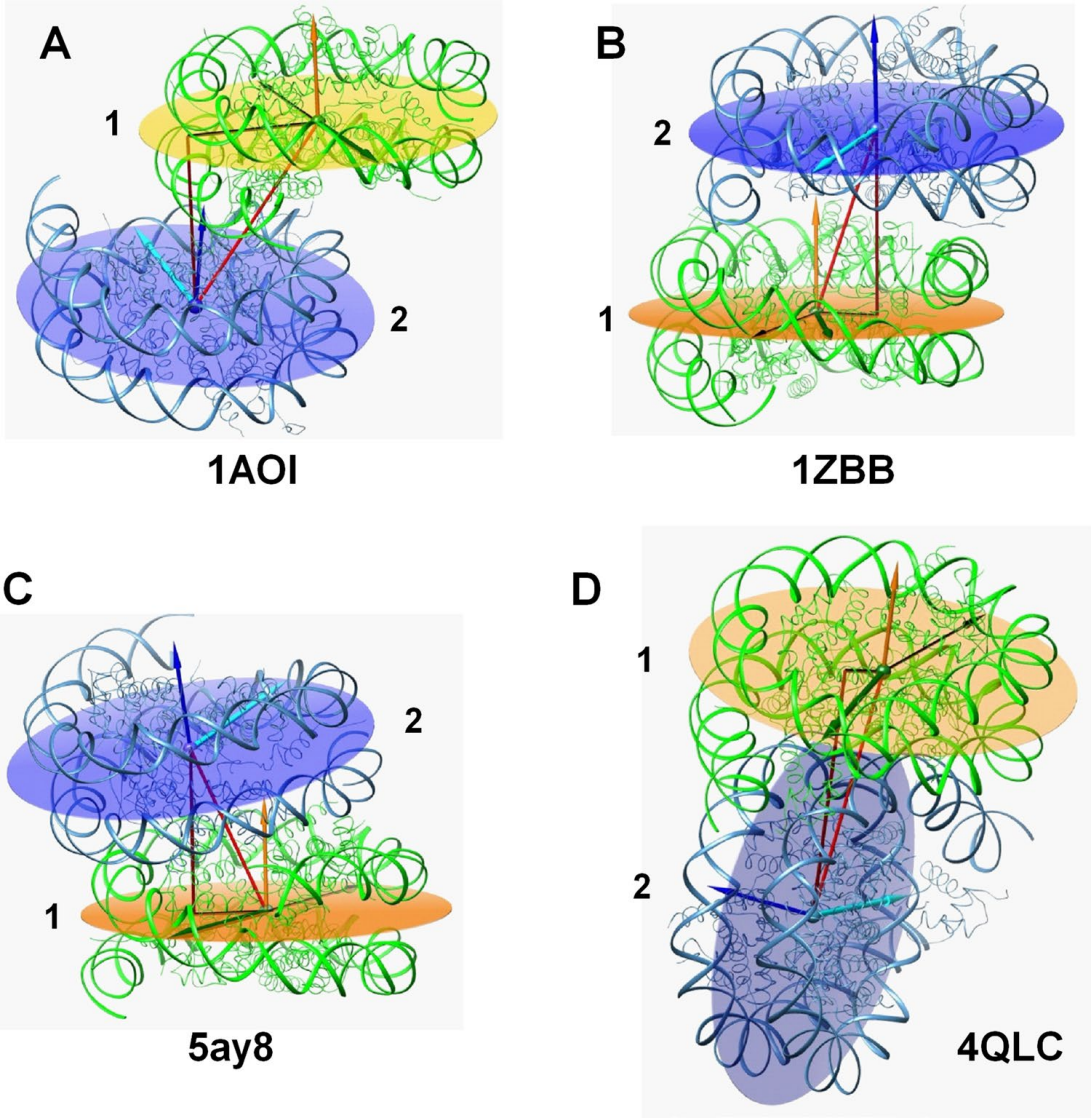
Examples of NCP-NCP contacts. (**A**) Typical NCP-NCP stacking observed in the published crystals. The 1AOI^3^ structure is used for illustration. (**B**) NCP-NCP stacking in the crystal of the tetranucleosome^24^ with the NCP-NCP distance 58.4 Å, shift 18.7 Å, rise 55.3 Å and head-to-head symmetry axes orientation (−25°). (**C**) Close NCP-NCP stacking observed in the NCP crystal with human H3.Y histone variant^61^ (NCP-NCP distance 54.2 Å, shift 21.6 Å, rise 49.7 Å, and 180° head-to-tail symmetry axes orientation). (**D**) One of the two NCP-NCP contacts in the crystal of the 167 bp chromatosome^51^. The angle between the NCP planes is equal to 85.5°, NCP-NCP distance is 77.2 Å, shift is 10.9 Å and 167° symmetry axes orientation. In all graphs, orange and blue planes and normal vectors indicate orientation of respectively the NCP1 and NCP2; green and cyan arrows on the planes show the positions of the symmetry axes; right-angle triangles are formed by lines connecting the centres of the NCP1 and NCP2, the projection of the NCP2 centre on the NCP1 plane and the NCP-NCP shift. The thin black arrow in the NCP1 plane shows the projection of the NCP2 symmetry axis on the NCP2 plane.

**Figure 6:**
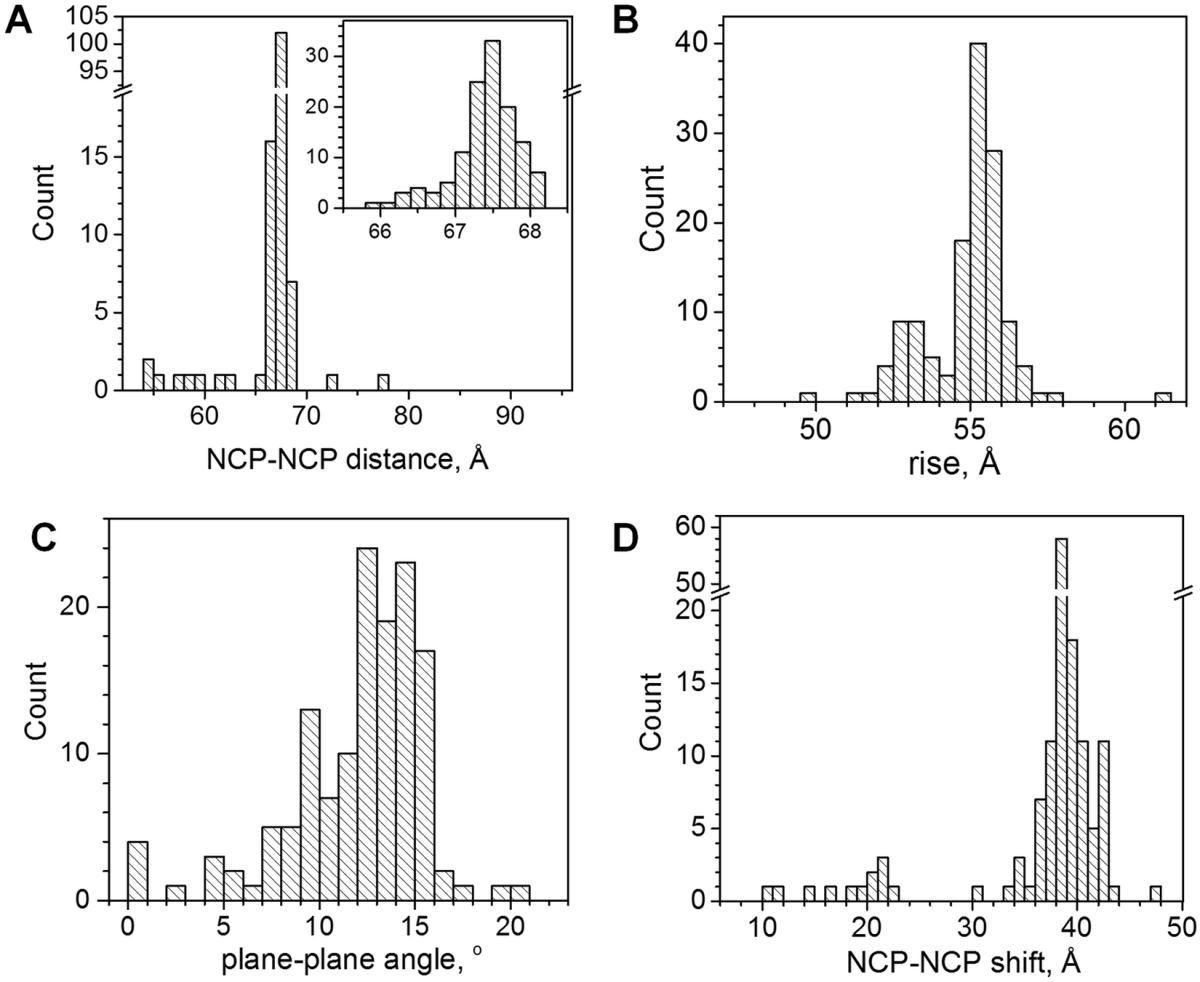
Parameters of NCP-NCP contacts in published crystal structures. (**A**) NCP-NCP distance. Inset shows data for the most populated range 66–69 Å. NCP-NCP distance exceeding 80 Å is excluded from statistics. (**B**) NCP-NCP rise (absolute values are given). (**C**) Angle between NCP planes. (**D**) NCP-NCP shift.

Using the approach described above we analysed the geometry of the NCP-NCP contacts in the published crystal structures. The results are presented in Figs 5 and 6 and in Supplementary Table S4. Figure 5 shows examples of the NCP-NCP contacts. The most typical case of NCP stacking observed in the vast majority of the crystals is depicted in Fig. 5A using the first atomic-resolution structure 1AOI^3^. The NCP-NCP distances are in the range 67–68 Å (Fig. 6A) and the absolute values of the rise between 54 and 57 Å (Fig. 6B). The plane-plane angle is in the range 8–16° (Fig. 6C) and NCP2 is tilted relative to NCP1 in the direction of the NCP1 symmetry axis (Supplementary Figure S3). In most NCP crystals the symmetry axes are oriented in a head-to-tail fashion (around ±180°; Fig. S1 and Supplementary Table S4; see also more discussion below). The shift varies from 38 to 44 Å (Fig. 6D). The NCPs shift is generally equal to two diameters of dsDNA substantially reducing the areas where the negative DNA surfaces of NCP1 and NCP2 are close to each other.

**Figure 7:**
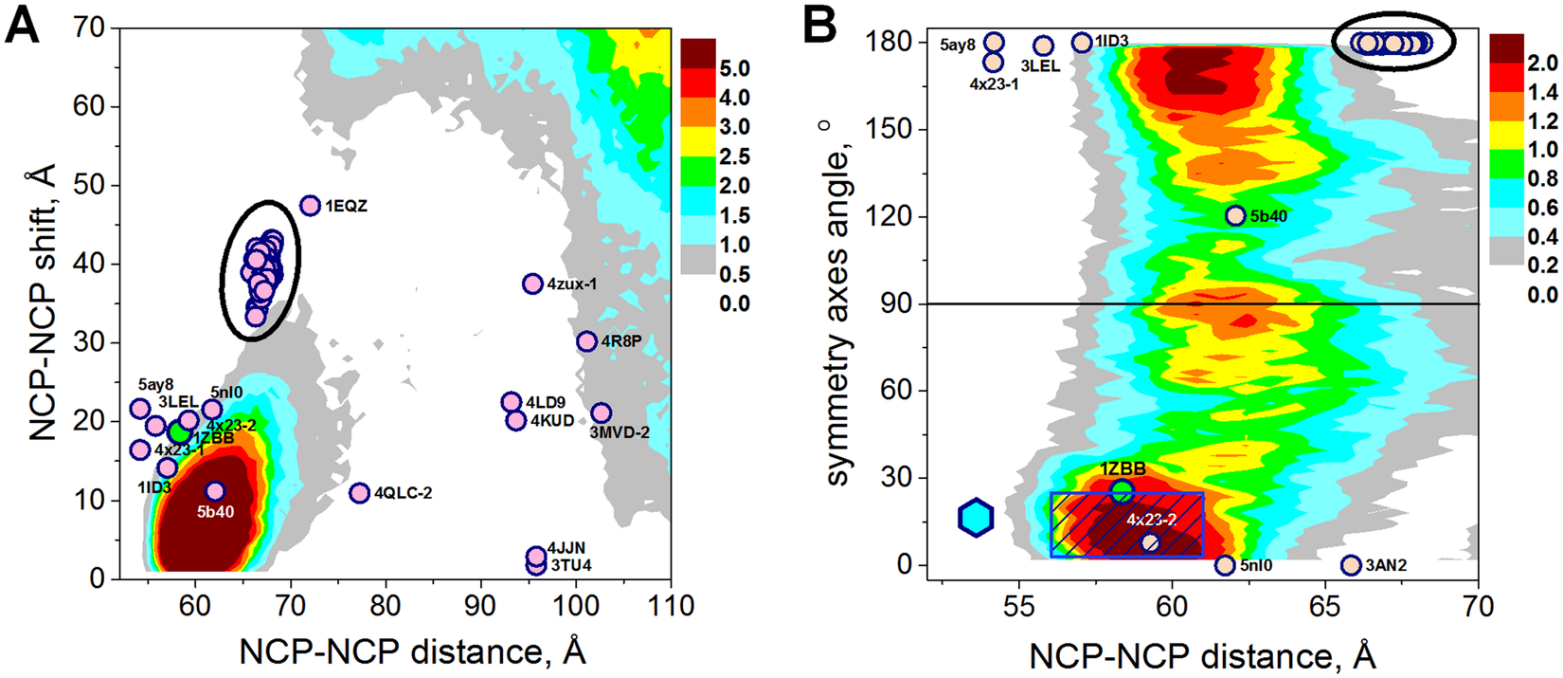
(**A**) Correlation between the NCP-NCP distance and the shift between NCP1 and NCP2. (**B**) Correlation between the NCP-NCP distance and the angle between the symmetry axis of NCP1 and the projection of the symmetry axis of NCP2 on the NCP1 plane. The contour areas in colour show the results of the CG simulations with CoHex^3+^ cations; points display the data calculated for NCP crystals and reported in Supplementary Table S3. Ovals highlight the areas where the data from most of the crystal structures are clustered.). Points that are outside the major clusters are labelled by pdb entry codes. Larger green points highlights tetranucleosome structure^24^, magenta hexagon point in (**B**) is for the one of the two NCP-NCP stacks in the 12–187 nucleosome array reported in the recent cryo-EM study^39^ Cyan hexagon point in (**B**) is for the NCP-NCP stacking in the 12–187 nucleosome array^39^, the shaded box illustrates the results for precipitated ordered NCP phases obtained from combined X-ray diffraction and cryo-EM studies^75^.

In Fig. 5B NCP-NCP stacking observed in the crystal structure of the tetranucleosome^24^ is displayed. This sort of NCP stacking is expected to be common in chromatin 30-nm fibres folded *in vitro* and *in vivo*. Here, the NCP-NCP distance is less than 60 Å, the rise is around 55 Å. The NCP-NCP shift is about 20 Å and roughly corresponds to the diameter of the dsDNA. The concertina-like packing of the nucleosomes in the two-start 30-nm chromatin fibre displays head-to-head positions of the NCP symmetry axes and this NCP orientation is expected to be very common. In general, very few structures with head-to-head axes orientation were reported: only three crystals (except the tetranucleosome) show a symmetry axis orientation around 0°; the NCP with the centromeric histone CENP-A^59^, an NCP complex with the centromeric protein CENP-C^60^, and 197-bp nucleosome in a complex with linker histone H1^38^ (Fig. 7B, Supplementary Table S4).

A few crystal structures have both rather small values of the shift (around 20 Å, Fig. 6D) and head-to-tail symmetry axis orientation. One of these structures, 5ay8^61^, is shown in Fig. 5C. The difference in the NCP-NCP shift between the 1AOI and 5ay8 crystals (about 20 Å smaller for the 5ay8) reduces overlapping between the NCPs in the crystal; compare Fig. 5A and C.

No NCP-NCP stacking is observed in the protein-NCP structures published to-date^50,53,62–69^. In these crystals the NCP-NCP distance exceeds 80 Å and the NCPs are separated from each other by proteins. The recently published structure of the 167 bp chromatosome, 4QLC^51^, also does not show NCP-NCP stacking. One of the two chromatosome-chromatosome crystal contacts is shown in Fig. 5D.

### NCP-NCP interaction investigated by coarse grained computer simulations

To reveal the major effects of electrostatic interactions on the attractive nucleosome – nucleosome interaction, we have recently developed a CG model that captures the major features of the NCP: the shape (volume excluded effect) of the dsDNA and the globular part of the HO; explicit representation of the charged groups (DNA phosphates, Lys, Arg, Glu and Asp amino acids) and flexible dynamic histone tails (Supplementary Methods and Figure S5A)^70–72^. CG Langevin MD simulations within a dielectric continuum model were performed for a systems containing ten^72^ and twenty^71^ CG NCPs in the presence of explicit mono- (K^+^), di- (Mg^2+^) or trivalent cobalt(III)hexammine^3+^ (CoHex^3+^, Co(NH_3_)_6_^3+^). Although CoHex^3+^ is not a biologically relevant cation, due to its symmetry and well-defined structure it is practical in modelling NCP aggregation *in vitro* and *in silico*. It was experimentally shown that the columnar hexagonal phase induced by the presence of this trivalent cation is the same as in the presence of the biological trivalent cation spermidine^3+ ^^73^. The results confirmed the experimental observations about the polyelectrolyte nature of the NCP and chromatin^74^ and showed that electrostatic forces make a decisive influence on the NCP – NCP interaction. In the presence of K^+^, NCPs repel each other, while in the presence Mg^2+^ cations the NCP-NCP interaction becomes slightly attractive, which is reflected by formation of small (2–4 NCPs) aggregates. The effective screening of the NCP negative charge in the presence of the tricationic CoHex^3+^ resulted in formation of a single aggregate both in systems with ten^72^ and twenty^71^ NCPs.

In the present work we further examine the results of the NCP + CoHex^3+^ simulations using the framework introduced above, focussing on an analysis of the internal structure of the aggregated NCPs. We compare structures of the NCP-NCP contacts observed in the CG simulation of 20 NCPs in the presence of CoHex^3+^ with observations in the NCP crystals, nucleosome arrays and NCP liquid crystalline phases. The results of the CG modelling reveals the contributions of the H4 and H2A N-termini in stabilisation of the NCP-NCP contact and allows making predictions about preferential orientations of the NCPs within the condensed phase.

Effective screening leads to formation of a single aggregate of NCPs formed in the presence of CoHex^3+^ (Supplementary Figure S5C) with NCP columns and some cases of ‘perpendicular’ conformations. The aggregation of the NCPs is dynamic with frequent dissociation/association of NCPs and the NCP-NCP distance distribution shows a stacking conformation at a maximum at about 60 Å (Supplementary Figure S5D).

Next we undertake a more detailed analysis of the NCP-NCP contacts observed in the aggregated NCP-CoHex^3+^ system and compare the simulation results with the available experimental data. For the CG NCP, the COM, the symmetry axis and the NCP plane of the coordinate system were calculated similarly to these parameters of the NCP atomic crystal structures as shown in Supplementary Figure S5B.

Figure 7 shows correlations between the NCP-NCP distance and the NCP-NCP shift (Fig. 7A) and the angle between the symmetry axes (Fig. 7B). Supplementary Figure S6 displays the correlation between the NCP-NCP distance and plane-plane angle. In these figures the coloured areas depict the density of the conformations observed in the CG simulations; circle points are for the NCP crystals, the hexagon point is for the cryo-EM structure of the nucleosome array^39^, the shaded box are for the electron microscopy (EM) and small angle X-ray scattering (SAXS) data obtained in studies of ordered NCP phases^75^.

Generally, for NCP-NCP contact distances below 75 Å, the the plane-plane angle is small (Supplementary Figure S6). Three groups of shift correlations can be observed: i) More than 100 structures have an NCP-NCP distance in the range 65–69 Å and a shift of 35–45 Å (highlighted by an oval in Fig. 7A). ii). A few crystals show both shorter NCP-NCP distance (54–62 Å) and a smaller shift (10–22 Å). iii) For the structures where the NCP was crystallized in complex with other proteins, NCP-NCP stacking is absent and the NCP-NCP distance is larger than 75 Å while the shift varies considerably.

In the CG simulations, the stacked NCPs are separated by 56–66 Å and shifted relative to each other by 0–15 Å showing a clearly defined area in the distance – shift correlation graph (Fig. 7A). The NCP-NCP shift is rather small and there are large areas of close DNA-DNA distances inside the NCP-NCP stack. Since both the electrostatic and the short range force potentials of the CG model are repulsive, we conclude that charge screening and ion-ion correlation contributions from bound CoHex^3+^ ions are efficient enough to establish a large area of NCP-NCP contacts between nucleosomes. The high efficiency of CoHex^3+^ in promoting condensation of DNA and chromatin has been well-established in a number of experimental studies^74,76^ (and references cited therein).

Figure 7B compares the correlation between the NCP-NCP distance and the mutual orientation of symmetry axes of the stacked NCPs (the angle δ in Fig. 4C). In most of the NCP crystals, the symmetry axes of the NCPs in the NCP-NCP stack are oriented in a “head-to-tail” fashion with the angle being very close to δ = ± 180°. One of the structures with monoubiquitinated histones H2B and H4^77^ (pdb 5b40) has δ = 120°. Only four crystal structures with stacked NCPs show “head-to-head” axis arrangement: The tetranucleosome^24^ (δ = −25°), the 197 bp chromatosome^38^ (δ = 0°) and the two NCP crystals with the centromere variant of the histone H3, CENP-A^59,60^ (δ = 0° and −8°; see Supplementary Table S4). The “head-to-head” axis orientation is statistically dominating inside the NCP aggregates in the CG simulations. This orientation is most common in the two-start 30-nm nucleosome fibres as it is observed in the tetranucleosome crystal^24^ and in the 12–167 array reported in the recent cryo-EM work^39^ (hexagon point in Fig. 7B; since atomic coordinates are not available the NCP-NCP distance and dyad-dyad angle cited in the paper are used). Furthermore, combination of SAXS and electron microscopy data shows that this co-linear positioning of the dyad axes is typical for ordered precipitated liquid crystalline NCP phases^73,75^ (shown as a shaded box in Fig. 7B). From the comparison of the NCP-NCP distance – axis angle correlation values it can be concluded that very good agreement is observed between the experimental and CG modelling data. However, the simulation data shows that the axis orientation can vary over a range of possible conformations with preference for the head-to-head and head-to-tail orientations. Interestingly, the simulation data displays a local maximum for the perpendicular orientation of the axes (δ around ±90°). Below we will analyse the contribution of electrostatic interactions and the role of the histone tails to the formation of the NCP and the NCP-NCP stacking.

### Analysis of ionic contacts in the NCP and NCP-NCP structures

The major component of the NCP, 147 bp dsDNA, carries a charge of −292 e and electrostatic forces make a major contribution both to the formation of the single NCP and to the NCP-NCP interaction. In order to analyse the histone tail interactions with DNA and the amino acids on the surface of the core, the HO amino acids were divided into two groups: the a.a. of the structured core domain and those of the histone tails. The charge content and the charge-charge contacts in the structured part of the NCP stacking were analysed. (Assignment of the tails is given in Supplementary Table S1 and in Fig. S7A; all other a.a. belong to the gHO). As a criterion of the formation of an ion-ion contact, a distance less than 7.5 Å between P atoms of DNA, CZ and NZ atoms of Arg and Lys; CD and CG atoms of Glu and Asp was defined.

Although the net charge of the gHO is positive (+52 e, Supplementary Table S1), the numerous contacts of the Lys and Arg with the DNA phosphates (respectively, 25 and 38) and with Asp, Glu carboxylates (18 and 38), lead to practically complete neutralisation of the positive charge of the gHO; so the surface of the NCP (DNA + gHO) is effectively negatively charged (Supplementary Figure S7B).

The NCP charged surface has two distinct negative patches formed on either side of the NCP cylinder by seven acidic a.a. (Glu56, 61, 64, 91, 92, Asp90 of the H2A and Glu110 of the H2B histones (Supplementary Figure S7B). This acidic patch is important for formation of chromatin secondary structures and for binding and transcriptional regulation of various nuclear proteins^52,78,79^. A similar analysis of the ion-ion interactions in the NCP has been carried out^80,81^ and our conclusions are in a general agreement with these earlier results. The large negative charge on the NCP surface necessitates screening in order to enable stacking between the NCPs, which would otherwise be energetically unfavourable. Clearly the histone tails that carry most of the positive charge of the HO make a significant contribution to this screening. Polyelectrolyte theory predicts that the tails must be electrostatically bound to the DNA practically at all physiologically relevant ionic conditions^74,82^. The H2A and H4 N-terminal tails are situated on the top and on the bottom of the NCP flat cylinder and these tails are particularly important for formation and stabilization of the NCP-NCP contact since they may be distributed between the two surfaces inside the stack. Notably, the H2A and H4 have similar length; number and distribution of charged Lys and Arg (see Supplementary Figure S7C). This similarity indicates that these tails might cooperate and exchange their contributions and positions in the NCP stacking contact.

**Figure 8:**
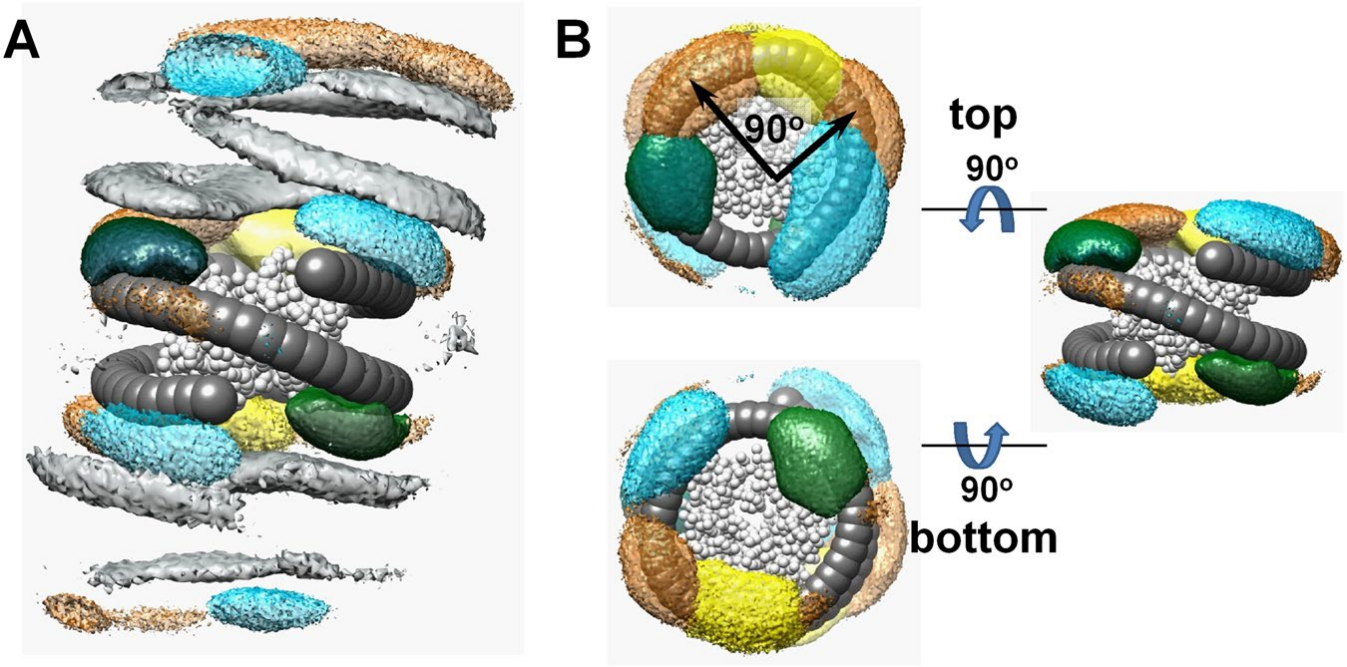
CG simulations reveal tail screening and tail-tail correlations in the NCP-NCP stacking contact. (**A**) Spatial distribution functions (SDF) showing the averaged particle densities around the central NCP (DNA, dark grey, HO, light grey) of DNA from the outside NCPs (grey), densities of the H4 and H2A N-termini belonging to the central NCP (H2A, yellow; H4, green) and external NCPs (H2A, yellow-orange; H4, cyan). (**B**) Detailed views of the internal and external SDFs of the H2A and H4 tails near the top and bottom surfaces of the central NCP. The colour scheme is the same as in (**A**) The top cartoon in (**B**) shows that there are two areas populated by the external H2A tail (indicated by black arrows). In (**A**) and (**B**), the beads of phosphates, H3, H2B tails, external HO and of CoHex^3+^ ions are not shown.

We combine data of the CG simulations with crystallography data to reveal contributions of the tails to the stabilization of the NCP-NCP stacking. Figure 8 summarises the results of the CG simulations concerning the participation of the H2A and H4 tails in stabilization of the NCP stacking. Figure 8A and B displays spatial distribution functions, SDFs (see Supplementary Methods for details on definition and calculation of the SDF) of the histone tails around a central NCP and illustrates how the H2A (yellow) and H4 (green) tails cooperatively shield the DNA-DNA repulsion in the stacked NCPs. The SDF gives a three dimensional picture of the averaged density of the particles of each of the tails around a central NCP and may distinguish between the contributions from the tails of the central NCP (internal) from the neighbour NCPs (external). The most common structural element is the columnar NCP stack with the four tails (one H2A and one H4 tail from each of the two NCPs) distributed between the surfaces of each NCP pair. The tails do not overlap and in Fig. 8B it is seen that one external H2A tail contacting the central NCP might occupy two different areas shifted relative to each other by about 90° (indicated in the figure by black arrows). Consequently, the necessity for a tail-tail cooperation in screening the NCP negative surfaces gives preference to the three distinct NCP-NCP orientations that correspond to the angles between the symmetry axes 0°, ±180° and ±90°. The head-to-head and head-to-tail positions are observed experimentally and in the crystal structure of the tetranucleosome^24^ and the H4 tails are located in the same areas as in the CG simulations (the coordinates of the H2A tails were not resolved experimentally).

Our CG simulations are based on a model that adequately describes electrostatic and excluded volume effects, which we believe are the most important factors that define the NCP-NCP interaction. The good agreement between the modelling data and available experimental information confirms the validity of this simplified approach. However, the present CG NCP model does not include specific factors such as hydrogen bonding, dipole-dipole and hydrophobic interactions that undoubtedly make important contributions to the NCP-NCP stacking. Specifically, in our simulations, binding of the H4 R17-R23 basic domain to the H2A/H2B acidic patch and the specific and exceptional influence of the H4 K16^83–86^ to the formation of folded chromatin structures are not adequately captured. It is reasonable to suggest that there exist multiply energetically favourable NCP stacked conformations and those that include the specific H4 tail – acidic patch binding may only contribute to a subset of all possible NCP-NCP conformations. While the role and contributions of the H4 tails to nucleosome – nucleosome interaction is widely appreciated and studied, investigation of similar contributions from the H2A N-termini that are revealed in the present CG simulations have so far not been observed experimentally. Another factor that is important for the NCP – NCP interaction but still did not receive much attention is the ability of the αC helix of the H2B histone that juts out of the HO surface to interact with various DNA and histone domains of the neighboring nucleosomes^87^.

In the majority of the crystals, overlap between the NCP surfaces in the stack is reduced due to the substantial shift (36–44 Å, Fig. 6D). The H2A and H4 tails seem to play important role in stabilisation of this conformation. Supplementary Table S5 lists the availability and binding modes of the H2A and H4 tails in the NCP crystal structures. The data shows that when the coordinates of at least some part of the tails are available, the H2A N-termini interact with the DNA of their own NCP and frequently with the DNA of the other NCP in the stack. One of the H4 tails binds to both their own DNA and to the acidic patch of the neighbouring NCP, while the coordinates of the second H4 tail are often unresolved (Supplementary Table S5). This illustrates the dynamic binding of this tail that can contribute to screening electrostatic repulsion from the DNA of the other NCPs in the crystal.

In most crystals, visualisation of the NCP1 – NCP2 stack shows two symmetrical internucleosome contacts between the histone (a.a. 101–122) and DNA (Supplementary Figure S8). Four lysine and a number of polar amino acids of the H2B αC helix can form ionic and hydrogen bonds with the DNA (as shown in Fig. S8) and this interaction as well as other possible inter-NCP H2B αC helix binding modes^87^ might contribute favourably to the formation of the NCP stack.

## Conclusions

In this work a simple and universal coordinate system based on the NCP atomic structure and its cylindrical symmetry was suggested. Our approach enables a numerical description of the relative NCP-NCP positions in condensed NCP or chromatin systems. Using this new NCP-centred coordinate system, a set of parameters was introduced to numerically characterise the mutual positions of the NCPs in the published crystal structures (Fig. 4).

An analysis of the NCP stacking revealed that in the most NCP crystals, the nucleosomes are in an almost perfect head-to-tail orientation (the angle between the symmetry axes is close to ±180°) and there is a significant shift of the NCPs relative to each other (36–44 Å). However, there are some examples of the head-to-tail orientation and a smaller (about 20 Å) shift; notably the tetranucleosome^24^ and the nucleosome array^39^. The dominance of the “large shift − head-to-tail orientation” structures might be caused by restrictions imposed by crystal packing, particularly the importance of the contacts between DNA ends. However, in nucleosome arrays with NRL less than 190 bp, folding into the two-start 30-nm fibre should lead to the head-to-head stacking. Furthermore, the two-start and interdigitated structures of the 30-nm chromatin fibre, also display the head-to-head orientation^26^. This might mean that *in vivo*, the head-to-head orientations are common. However, it is also likely that the head-to-tail stacking observed in the crystals might be frequent *in vivo* where melted liquid-like state of chromatin^32^ facilitates in-trans interdigitated contacts between the nucleosomes.

The results of the CG MD simulations are in good agreement with structural data obtained for the NCP crystal structures, NCP liquid crystalline phases and for the nucleosome arrays. The CG MD simulations give valuable insights on the nature of nucleosome interaction and demonstrate that electrostatic interactions plays a decisive role not only in the general phase behaviour of chromatin/nucleosome systems but also is essential for specific structural arrangements inside condensed chromatin. The simulations revealed the novel finding that correlations between the H2A and H4 N-terminal tails are important for shielding DNA repulsion in the stacked NCPs. The areas sampled by these tails do not overlap and make the head-to-head and head-to-tail NCP orientations more populated than the other stacking arrangements.

## Methods

### Analysis of NCP and NCP-NCP stacking structures

Structures of the nucleosome core particles were downloaded from the Protein Data Bank (PDB; http://www.rcsb.org/pdb/) maintained by Research Collaboratory for Structural Bioinformatics^88^. Since there is no convention about naming and numbering of the histone proteins and amino acids as well as DNA strands and nucleotides, every pdb structure was analysed individually using home-written Fortran scripts. The Chimera software^89^ was used to build molecular structures and surfaces and to create figures. Structural parameters of the NCP double stranded (ds) DNA were calculated by Curves+^90,91^. Parameters of the DNA superhelix (radius, pitch, number of DNA base pairs in one turn, dyad axis) were determined using coordinates of the 129 central dsDNA axis points (of total 145–147 bp) and fitting them to an ideal superhelix using a modified approach developed by Kahn^92,93^. The details of this procedure are given in the Supplementary Material. The choice of this particular number of the DNA base pairs for the SH fitting is based on the detailed analysis by Richmond and Davey^56^ who excluded a few bp at the entry/exit of the NCP since these dsDNA stretches exhibit reduced bending and detachment from the HO surface. We apply this standard 129-point frame to define the parameters of all reported NCP structures (except one case where less than 129 bp coordinates were reported)^59^. In the Results and Discussion we show that using this 129 bp length does reflect a real division of the nucleosomal DNA into a tightly bound central and a looser entry/exit domain.

Structures of the stacked NCPs were obtained by making copies of the single NCP according to its crystal packing and selecting the pair of closest NCPs. In a few cases when the NCP was crystallized in a complex with another protein, close NCP-NCP stacking was absent or several NCP-NCP arrangements were possible. These cases were also analysed. NCP-NCP parameters were not determined for PDB structures that contain incomplete NCP^69^, or obtained by cryo-EM method^53,54^.

All the scripts used for the analysis in the present work is available from the authors to be shared with interested users.

### Coarse-grained MD simulations of NCP-NCP interaction

Detailed description of the CG NCP model, force field and setup of the MD simulations as well as methods of analysis of the MD data is given in our earlier work^71,72^ and in the Supplementary Methods.

Briefly, the NCP model included 1350 particles and consisted of a CG model of DNA with resolution of 5 particles per two DNA bp (one central bead for four nucleosides forming 2 bp and four beads representing phosphate groups). We used a CG model of the HO core and histone tails with one site per each a.a. DNA consisting of 74 such units, modelling 148 bp, was wrapped around the histone core. The DNA structure was maintained by harmonic bond and angle potentials; the beads of the gHO were placed according to the 1KX5 crystal structure and the integrity of the HO was maintained by applying an elastic network scheme^94^. The histone tails were modelled as 10 strings of linearly-connected beads of length and a.a. sequence according to the 1KX5 structure^4^. Electrostatic interactions were treated explicitly by a Coulombic potential and using a dielectric continuum description of the solvent water, assigning unit charges to the phosphate beads of the DNA and charged a.a. (Lys, Arg, Glu and Asp). The net charge of the CG NCP was −150 *e*, with DNA, gHO and histone tails carrying respectively −296 *e*, +52 *e* and +94 *e* charge. This CG model of the NCP and description of the CoHex^3+^ ion has been thoroughly validated in our previous work and describes adequately a range of experimental observations and data both quantitatively and qualitatively^71,72^.

Radial (RDF) and spatial (SDF) distribution functions were calculated using scripts described in our earlier simulation work^72,95,96^. ESPResSO software^97^ was used to run the simulations.

### Data availability statement

The datasets (NCP structures) analysed during the current study are available in the RCSB protein data bank repository: http://www.rcsb.org/pdb/home/home.do. The datasets (Langevin MD simulations) generated and analysed during the current study are available from the corresponding author on reasonable request.

## Electronic supplementary material

Supplementary Information
