# Supplementary material for: A systematic analysis of nucleosome core particle and nucleosome-nucleosome stacking structure
Source: Sci Rep. 2018 Jan 24;8:1543. doi: 10.1038/s41598-018-19875-0 (PMC5784010; doi:10.1038/s41598-018-19875-0)
Supplement: Supplementary file 1 — Supplementary Information [file 41598_2018_19875_MOESM1_ESM.pdf]

Supporting Material for the manuscript:

"A systematic analysis of nucleosome core particle and nucleosome-nucleosome stacking structure"

by Nikolay Korolev, Alexander P. Lyubartsev, and Lars Nordenskiöld

I. Supporting computations methods

1.1. Calculation of the orientation and parameters of an ideal superhelix

The procedure to determine an ideal superhelix representing dsDNA wrapped around the histone core is performed as follows: First, for each of the NCP crystal structures reported in the PDB database, a 129 bp central fragment of DNA wrapped around octamer core was selected and the coordinates of the axis points of the double helical DNA were determined at the level of each basepair using the Curves+ software (1,2). (See ref (1) for definition and description of the calculation algorithm of the DNA axis).

Let us denote axis the coordinates as $\{\vec{r_i}\}$, i = 1,...,129. We define the DNA superhelix axis as the axis most "perpendicular" to all the fragments of the dsDNA axes, by minimization of the sum of scalar products of vectors representing the DNA axis, $\vec{r}_{i+1} - \vec{r_i}$, with a unit vector $\vec{A}$ directed along the superhelix axis:

$$S = \sum_{i=1}^{128}\left((\vec{r}_{i+1} - \vec{r_i}) \cdot \vec{A}\right)^2 \qquad \text{(S1)}$$

Minimization of S varying the coordinates of the vector $\vec{A}$ under constraint condition $\vec{A}^2 = 1$ leads to equation:

$$\frac{d}{d\vec{A}}\left(S - \lambda\vec{A}^2\right) = 0 \qquad \text{(S2)}$$

where λ is a Lagrange multiplier. Further mathematical operations result in an eigenvalue problem:

$$M\vec{A} = \lambda\vec{A} \qquad \text{(S3)}$$

with the matrix $\boldsymbol{M}$ determined by the tensor products of vectors:

$$M = \sum_{i=1}^{128}(\vec{r}_{i+1} - \vec{r_i}) \otimes (\vec{r}_{i+1} - \vec{r_i}) \qquad \text{(S4)}$$

Solution of the eigenvalue problem (eq. S3) results in 3 eigenvalues and 3 eigenvectors. The direction of the superhelix axis is determined by the eigenvector corresponding to the minimum eigenvalue. Furthermore, the eigenvector corresponding to the maximum eigenvalue determines direction of the dyad axis while the third eigenvector is orthogonal to the two first. The three eigenvectors thus determines the local coordinate system of the NCP in which the superhelix axis determines the Z direction and dyad axis determines X direction.

After determination of the superhelix axis and the NCP local coordinate system, the parameters of the superhelix can be determined straightforwardly. The coordinates of the dsDNA axis $\{\vec{r_i}\}$ are recalculated in a local cylindrical coordinate system as (r'_i, ϕ_i, h_i). The average of the radial coordinates r'_i determines the superhelix radius; the average phase shift

(ϕ_{i+1} - ϕ_i) determines the average number of base pairs per superhelix turn, and the average difference of the height coordinates (h_{i+1} - h_i) multiplied by the number of base pairs per superhelix turn defines the superhelix rise.

1.2. Corse Grained Molecular Dynamics simulations of NCP condensation

To get a deeper insight into the structure of the NCP-NCP stacking we analysed the results of our earlier coarse-grained (CG) MD simulations in a system of 20 CG-modelled NCPs in the presence of explicit mono- (K^+), di- (Mg^{2+}) and tri- ($CoHex^{3+}$ = $[Co(NH_3)_6]^{3+}$) cations (3,4). The detailed description of the CG NCP model, construction of the simulation system and explanation of the MD simulation setup and analysis of the results are given in the cited works (3,4). Below brief overview of the NCP model and applied MD method is given.

1.2.1. Coarse-grained model of the NCP. A CG NCP model reproduces shape of the real NCP combining histone octamer core and double helical DNA wrapped around it. It also includes flexible histone tails with resolution of 1 particle per amino acid (see Fig. 7A of the main text).

The 148 bp of the nucleosomal DNA are described as sequence of 74 five-bead units where each unit includes four beads each representing DNA charged phosphate group and one bead resembling four nucleosides from the two DNA base pairs. The internal DNA structure was maintained by bond and angular interactions fixed between neighbouring CG sites by harmonic potentials. Each phosphate group was assigned a charge –1e. The overall DNA structure is similar to space-filling grooved B-form DNA model with explicit charged on the phosphate groups used previously to study the ionic ion-DNA interaction and selectivity (5-10).

In the core part of the histone octamer (HO) each amino acid was represented as a bead with a charge assigned according to the amino acid type: lysine and arginine, +1e; aspartate and glutamate, –1e; the other amino acids were neutral. The initial coordinates of the beads (of total number 710; with 116 positive, 64 negative, and 530 neutral particles; net charge +52e) were set to the centers of mass of the amino acids determined from the NCP high resolution crystal structure, 1KX5 (11). In order to keep the structure of the core region intact during simulations, .the particles of the histone core were bound applying an elastic network scheme (12). The DNA was linked to the HO core by harmonic bonds connecting each DNA central bead with the nearest amino acid bead of the histone core according to the 1KX5 crystal structure. The histone tails were modeled as 10 strings of linearly-connected beads with the length and charge assignment mimicking the *Xenopus laevis* histones used in the

1KX5 crystal (11). The number of particles in each tail was 20, 35, 43, 24 and 13 for the H2A, H2B, H3 H4 and H2A-C (H2A-C is the short unstructured tail at the C-terminal of the H2A histone). The charges were assigned according to the amino acid sequence, resulting in total charge +94e from the 10 histone tails. The particle of each tail closest to the histone core was connected to the HO core according to the histone amino acid sequence.

The total number of CG beads comprising each NCP was 1350. The short range interactions were described by a repulsive r^{-12} potential with effective radii specific for each CG site so that contact distances between particles matched the ones observed in the NCP crystal structures and in the NCP (13,14) and in the histone tail – DNA MD simulations (15-17). The charges of the DNA, histone core and histone tails as well as of ions were treated explicitly by a Coulombic potential scaled by the dielectric permittivity of water $\varepsilon = 78$. The net charge of each NCP was $-150e$, with contributions $-296e$, $+52e$ and $+94e$ from DNA, histone core and histone tails, respectively.

1.2.2. Langevin Molecular Dynamics. The Langevin MD method as implemented in the ESPResSo package (18) was used for the simulations (see (3,4) for details). The friction and random forces mimic the effect of solvent, which makes the method very suitable to simulate continuum solvent models (19,20) . Since we are interested only in equilibrium properties, the masses of the particles and the parameters of the Langevin thermostat were set to get fast sampling of the configuration space and an efficient computation of the canonical averages. The electrostatic interactions were treated by the P3M method (21).

In the present work, a cubic periodic box of size 500 Å contained 20 CG NCPs and 1000 neutralizing three-valent counterions representing $CoHex^{3+}$. The effective $CoHex^{3+}$ radius was set to 3.5Å (which was the same as in the earlier simulations with 10 NCPs (3) and was validated in our previous work (8)). The time step was set to 0.04 in the reduced units used in ESPResSo package (which correspond to about 30 fs in real units). Simulations were started from a random configuration, were run for 2×10^{7} time units (equivalent to 15 μs of real time) of which the first 10^{7} steps were disregarded in analysis. The obtained trajectories were used for computations of RDFs. Convergence of the simulations was checked by calculations of NCP-NCP center of mass RDFs in $2.5\cdot10^{6}$ MD steps windows and ensuring that no trends in RDFs are observed.

One of the major advantages of the models developed in (3) is that the systems of one or multiply NCPs provide an accurate description of the electrostatic interactions, which includes mobile ions, charges of DNA phosphate groups as well as charges of the histone

tails and in the core region. The beads belonging to the DNA and the histone octamer are held together in a single NCP by a network of harmonic bond and angle interactions.

1.2.3. Spatial Distribution Function (SDF). The density distribution of mobile species (ions or particles of the histone tails) as well as other NCPs around a given NCP can be visualized by calculation of spatial distribution functions (SDF) using an approach developed earlier in MD simulations of DNA (22-24). The local coordinate frame for the SDF was defined by the positions of three particles chosen in the distant parts of the gHO. The SDF density of every set of particles (e.g. belonging to one of the tails) was calculated in this frame which was also used for calculation of the average NCP structure. SDFs were visualized as iso-surfaces drawn at the given level of intensity around the central NCP.

II. Supporting Figures

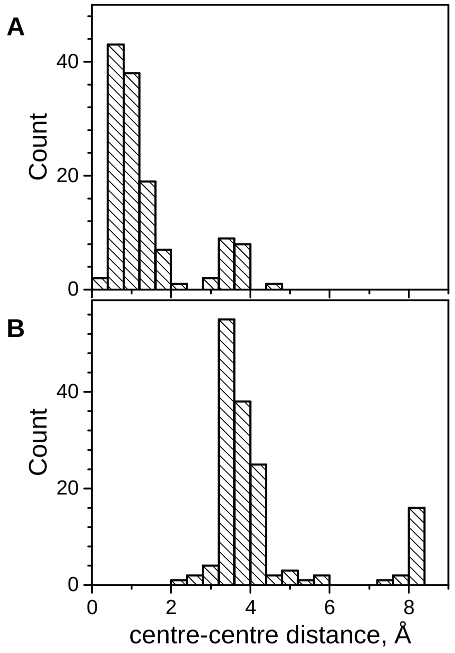

Figure S1. Statistics of distances between centres of the two coordinate systems: one based on the NCP symmetry proposed in this work; the other using the trace of the DNA helical axis wrapped as superhelix (SH) around the histone octamer. (**A**) The origin of the SH-based system is defined using all 145-147 DNA helical axis points. (**B**) The origin of the SH-based system is defined as the centre of an ideal SH found by fitting the coordinates of the 129 central DNA axis points (centre-centre distances shown in (B) are listed in the last column of Table S2 of the SM).

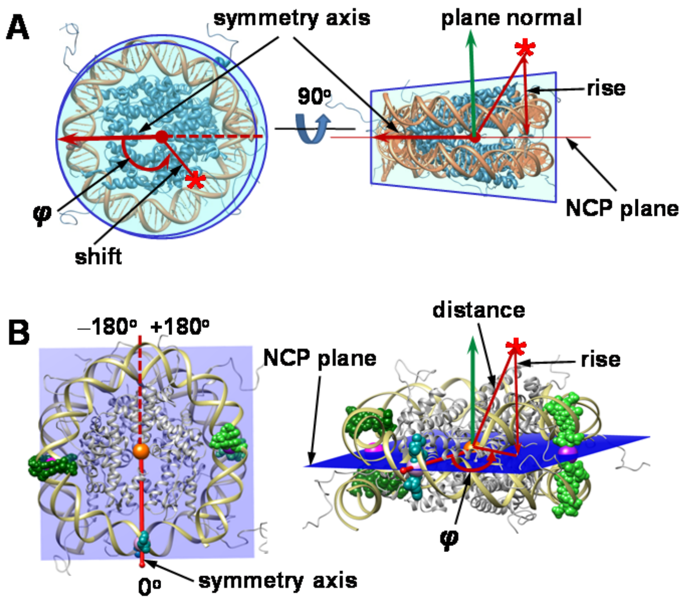

Figure S2. The set of parameters defining how the position of an arbitrary point (red star) is described in the NCP-centred coordinate system. (**A**). NCP approximated as wedge cylinder shown from the top (left) and from the side (right). The position of a point in the NCP coordinates is defined by (i) distance to the NCP centre (COM of the gHO); (ii) shift, the distance from the gHO COM to a projection of the point on the NCP plane; (iii) shift angle φ, the angle between the NCP symmetry axis and the shift line (**Fig. S2A**, left); (iv); rise is a distance from a point to the NCP plane. The value of the rise may be positive or negative depending on the point being located above or below the NCP plane. (**B**). Additional explanation on the NCP coordinate system. The left cartoon shows the range of the angle φ changing from 0° to respectively +180° or –180° to the right or to the left from the symmetry axis. The right cartoon shows that the distance from the point to the NCP centre may also be used.

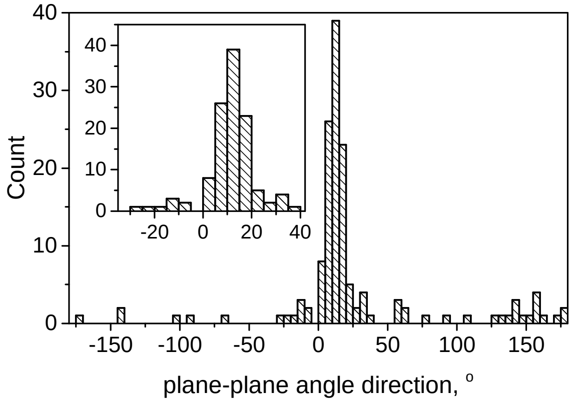

Figure S3. Statistics of direction of the angle between planes of the two NCPs (tilt direction). The tilt direction is defined as an angle between symmetry axis of the NCP1 and projection of the vector that is normal to the NCP2 plane as explained in Fig. 6D of the main text. Insert shows the data for the most populated range in the published crystal structures. Numerical data are given in Table S3 of the Supporting Material.

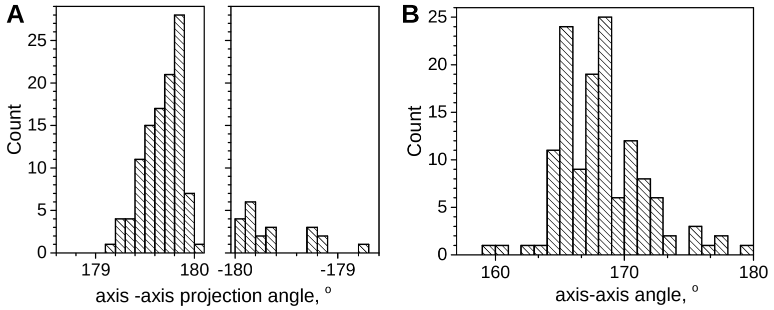

Figure S4. Statistics of mutual orientation of the symmetry axes for the stacking contact between NCPs in the published crystal structures. (**A**) Axis-axis orientation or angle between symmetry axis of the NCP1 and projection of the symmetry axis of the NCP2 on the NCP1 plane (see scheme in Fig. 5B of the main text). (**B**) Axis-axis angle. In (**A**) and (**B**) only range of angles characteristic for the head-to-tail NCP orientation is included; few cases of the alternative head-to-head axis orientations are not shown. Numerical data are given in Table S3 of the Supporting Material.

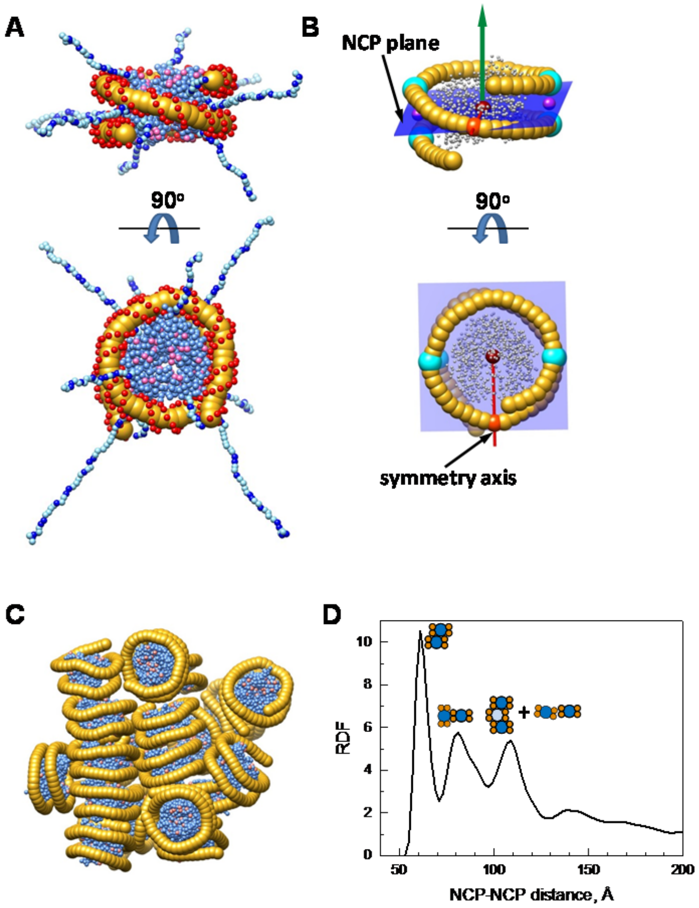

Figure S5. Coarse graining modelling of NCP-NCP interactions. (**A**) CG model of the NCP with explicit charges on the DNA and histones. Front and top views of the model are shown in the top and bottom panel, positively charged amino acids of the tails are in blue; negatively charged particles of the core histone domain are in pink. (**B**) Schematic representation of the symmetry coordinates developed for the CG NCP. The COM (red sphere) was defined using the coordinates of the amino acid beads of the core HO; the symmetry axis (red rod) was drawn through the COM and the DNA central particle The NCP plane (shown in blue) was defined as the median plane of the two planes which had a common line, the symmetry axis, while the third points of these two planes was placed in the middle of the line connecting two DNA beads of the two opposite DNA strands at the opposite sides of the NCP dyad axis (the vector normal to the plane is a green arrow). DNA beads used to define symmetry axis and plane are highlighted in red and cyan. (**C**) A snapshot of the simulation in the system of 20 CG NCPs with $CoHex^{3+}$ cations. Histone tails and $CoHex^{3+}$ cations are not shown for clarity. (**D**) NCP-NCP distance correlation calculated for the system of 20 CG NCPs with $CoHex^{3+}$ cations (adapted from (4) with permission). Cartoons near the peaks sketch the respective structures of the NCP-NCP contact.

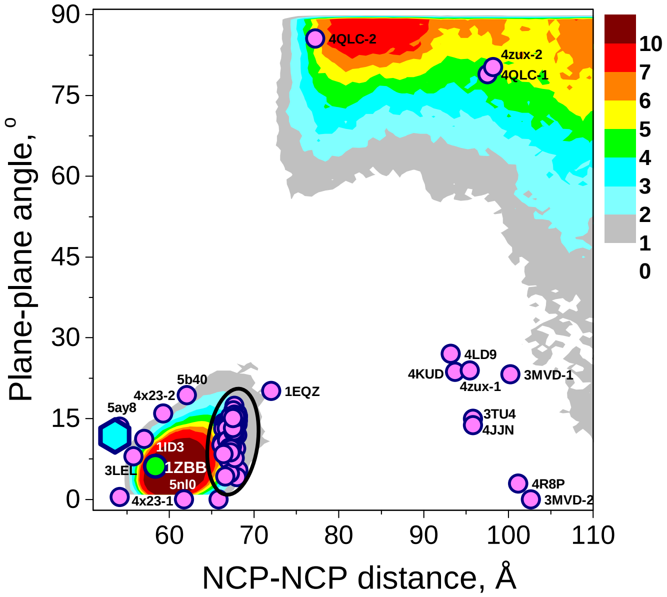

Figure S6. Correlation between the NCP-NCP distance and angle between the NCP planes. Contour areas in colour are the result of the MD simulations for 20 NCPs with $CoHex^{3+}$ cations; points display the data calculated for the NCP crystals and reported in Table S3 of the SM. Oval highlights the area where the data from most of the crystal structures are clustered (points overlap each other). Points that are outside the major cluster are labelled by pdb entry code. Larger green point highlights tetranucleosome structure (25); magenta hexagon point is for the one of the two NCP-NCP stacks in the 12-187 nucleosome array reported in the recent cryo-EM study (26); this point is not calculated using approach proposed in the present work but plotted using the values reported in the cited paper. Points in the range of NCP-NCP distance 80-110 Å are obtained for the NCP-protein crystals where NCPs are separated from each other.

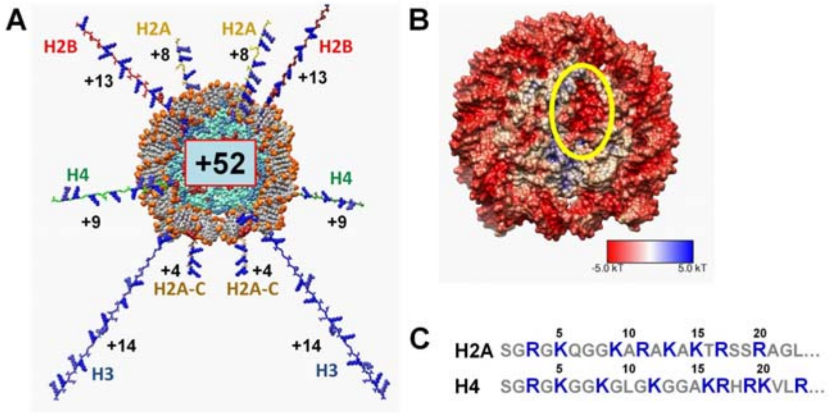

Figure S7. The NCP as a negatively charged polycation-polyanion complex. (**A**). Distribution of the basic amino acids in the NCP. The histone tails (shown in their stretched conformation) carry the major part of the positive charge (indicated for each tail); the net charge of the HO globular part is shown in cyan box. (**B**). The electrostatic potential of the NCP calculated for its structured part (tails are excluded). The location of the H2A/H2B acidic patch is indicated by a yellow oval. The adaptive Poisson-Boltzmann Solver (27) included into the Chimera visualization software (28) was used to calculate the surface potential using the following parameters: temperature 298 K, solvent radius 1.4 Å, 150 mM KCl. (**C**). The sequence of the H2A and H4 histone tails. Positively charged Lys and Arg are highlighted by larger blue font.

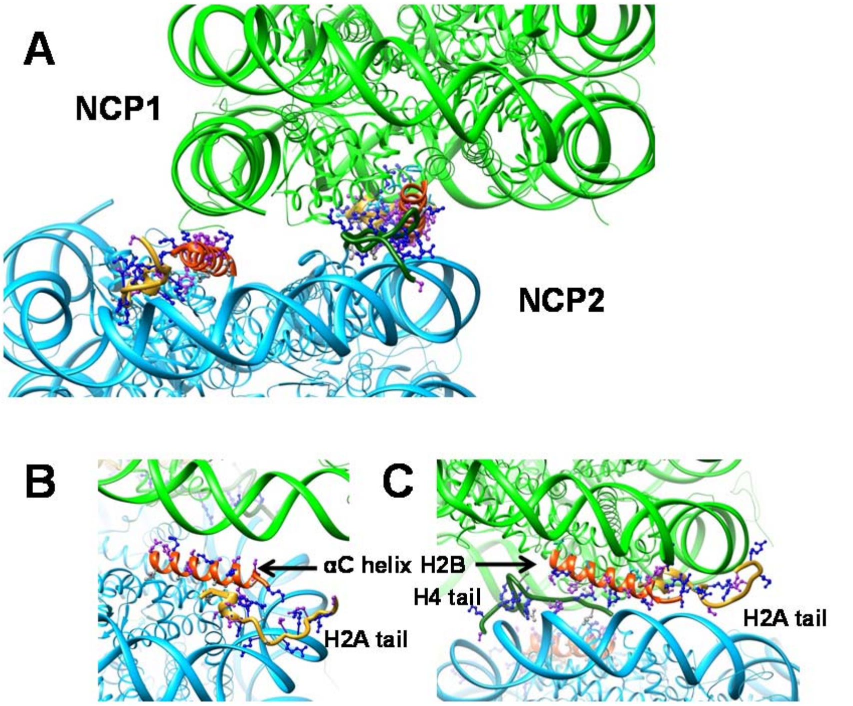

Figure S8. Most frequent structural arrangement of screening DNA-DNA repulsion between stacked nucleosomes in the NCP crystals. The 1KX5 structure (11) was used for illustration. (**A**). There are two areas where dsDNA of the two stacked NCPs (NCP1 is green, NCP2 is sky blue) are close to each other and in both cases the αC helices of the H2B histones (shown in orange) are located between the DNA surfaces. (**B**). Structure of the DNA-DNA contact shown in (**A**) on the left hand side. In addition to the H2B αC helix of NCP2 which is close to the DNA of NCP1, N-terminal of the NCP2 H2A histone (coloured yellow) screens the DNA of its own NCP. (**C**). Structure of the DNA-DNA contact shown in (**A**) on the right hand side. The H2B αC helix of NCP2, the histone tails of H2A (from NCP1, shown in yellow) and H4 (from NCP2, dark green) take part in screening DNA-DNA repulsion. The H2A (NCP1) and H4 (NCP2) tails interact with the DNA of its own NCP. In all cartoons, side chain atoms of the amino acids belonging to the highlighted histone domains are shown with atoms of positive lysine and arginine a.a. coloured blue and polar a.a. in purple. Note, that only part of the atoms in the histone tails of the 1KX5 structure has been assigned non-zero occupancies; and atoms of many residues at the ends of the N-termini have high thermal factors. Zero occupancy and/or high thermal factor both indicate dynamic nature of the tails in the NCP crystals.

III. Supporting Tables

Table S1. Charged amino acids in the histone octamer, $(H3/H4)_2$ tetramer, H2A/H2B dimer, in the histone structured domain (core) and in the tails.

Histones	Number of a. a.	Net charge (Lys, Arg; Asp, Glu)	Charge per a. a.
Octamer	974	+148 (+114,+106; –24,–48)	+0.15
Tetramer $(H3/H4)_2$	474	+76 (+48,+64;–14,–22)	+0.16
Dimer (H2A/H2B)	250	+36 (+33, +21, –5,–13)	+0.14
Structured core	712	+52 (+46,+72; –22,–44)	+0.073
Histone tails	262	+96 (+68, +34, –2, –4)	+0.37
H2A (1–20)	20	+8 (+4, +4; –0, –0)	+0.40
H2A-C (117–128)	12	+4 (+5, +0; –0, –1)	+0.33
H2B (1–33)	33	+13 (+12, +3; –1, –1)	+0.39
H3 (1–43)	43	+14 (+8, +6; –0, –0)	+0.33
H4 (1–23)	23	+9 (+5, +4; –0, –0)	+0.39

Table S2. Basic information about published crystal structures with containing nucleosome core particle.

No	PDB code	Reso-lution, Å	DNA	Histones	Notes	Refe-rence
1	1AOI	2.8	146 bp, α-satellite	*X. laevis*		(29)
2	1EQZ	2.5	146 bp, palindromic	Erythrocytes, *G. gallus*		(30)
3	1F66	2.6	146 bp, palindromic	H2A.Z, *H. sapiens*; H2B, H3, *X. laevis*; H4, *M. musculus*		(31)
4	1ID3	3.1	146 bp, palindromic	*S. cerevisiae*		(32)
5	1KX3	2.0	146 bp, palindromic	*X. laevis*		(11)
6	1KX4	2.6	146 bp, palindromic	*X. laevis*		
7	1KX5	1.94	147 bp, palindromic	*X. laevis*		
8	1M18	2.45	146 bp, palindromic	*X. laevis*		(33)
9	1M19	2.30	146 bp, palindromic	*X. laevis*		
10	1M1A	2.65	146 bp, palindromic	*X. laevis*		
11	1P34	2.7	146 bp, α-satellite	*X. laevis*		(34)
12	1P3A	3.0	146 bp, α-satellite	*X. laevis*		
13	1P3B	3.0	146 bp, α-satellite	*X. laevis*		
14	1P3F	2.9	146 bp, α-satellite	*X. laevis*		
15	1P3G	2.7	146 bp, α-satellite	*X. laevis*		

16	1P3I	2.3	146 bp, α-satellite	*X. laevis*		
17	1P3K	2.9	146 bp, α-satellite	*X. laevis*		
18	1P3L	2.4	146 bp, α-satellite	*X. laevis*		
19	1P3M	2.9	146 bp, α-satellite	*X. laevis*		
20	1P3O	2.75	146 bp, α-satellite	*X. laevis*		
21	1P3P	2.7	146 bp, α-satellite	*X. laevis*		
22	1S32	2.05	146 bp, α-satellite	*X. laevis*		(35)
23	1U35	3.0	146 bp, α-satellite	H2B, H3, H4 *M. musculus;*macroH2A *H. sapiens,*		(36)
24	1ZBB	9.0;	4-167-601 sequence	*X. laevis*	tetrasome	(25)
25	1ZLA	2.9,	146 bp, α-satellite	*X. laevis*	LANA protein	(37)
26	2CV5	2.5	146 bp, palindromic	*H. sapiens*;		(38)
27	2F8N	2.9	146 bp, α-satellite	H3, H4 *X. laevis;* H2B *M. musculus*; macroH2A *H. sapiens,*		No ref
28	2FJ7	3.2	Synthetic, 16 A/T bp	*X. laevis*		(39)
29	2NQB	2.3	146 bp, α-satellite	*D. melanogaster*		No ref
30	2NZD	2.65	145 bp, 601	*X. laevis*		(40)
31	2PYO	2.43	147 bp, α-satellite	*D. melanogaster*		(41)
32	3A6N	2.7	146 bp, synthetic	*H. sapiens*	testis specific H3	(42)
33	3AFA	2.5	146 bp, synthetic	*H. sapiens*		
34	3AN2	3.6	147 bp, synthetic	*H. sapiens*	CENP-A	(43)
35	3AV1	2.5	146 bp, palindromic	*H. sapiens*;		(44)
36	3AV2	2.8	146 bp, palindromic	*H. sapiens*;		
37	3AYW	2.9	146 bp, synthetic	*H. sapiens*	H3K56Q	(45)
38	3AZE	3.0	146 bp, synthetic	*H. sapiens*	H3K64Q	
39	3AZF	2.7	146 bp, synthetic	*H. sapiens*	H3K79Q	
40	3AZG	2.4	146 bp, synthetic	*H. sapiens*	H3K115Q	
41	3AZH	3.49	146 bp, synthetic	*H. sapiens*	H3K122Q	
42	3AZI	2.7	146 bp, synthetic	*H. sapiens*	H4K31Q	
43	3AZJ	2.89	146 bp, synthetic	*H. sapiens*	H4K44Q	
44	3AZK	3.2	146 bp, synthetic	*H. sapiens*	H4K59Q	
45	3AZL	2.7	146 bp, synthetic	*H. sapiens*	H4K77Q	
46	3AZM	2.89	146 bp, synthetic	*H. sapiens*	H4K79Q	
47	3AZN	3.0	146 bp, synthetic	*H. sapiens*	H4K91Q	
48	3B6F	3.45	147 bp, palindromic	*X. laevis*	cisplatin	(46)
49	3B6G	3.45	147 bp, palindromic	*X. laevis*	oxaliplatin	
50	3C1B	2.2	146 bp, α-satellite	*X. laevis*	H3K79Me2	(47)
51	3C1C	3.15	146 bp, α-satellite	*X. laevis*	H4K20Me3	
52	3KUY	2.9	145 bp, 601	*X. laevis*		(48)
53	3KWQ	3.15	146 bp, α-satellite	*X. laevis*	H3K56Q	(49)
54	3KXB	3.2	146 bp, α-satellite	*X. laevis*	H3K56Q	
55	3LEL	2.95	147 bp, α-satellite, modified	*X. laevis*		(50)
56	3LJA	2.75	147 bp, α-satellite, modified	*X. laevis*		(51)
57	3LZ0	2.5	145 bp, 601	*X. laevis*		(52)

58	3LZ1	2.5	145 bp, 601	*X. laevis*		
59	3MGP	2.44	147 bp, α-satellite	*X. laevis*	Co2+	(53)
60	3MGQ	2.65	147 bp, α-satellite	*X. laevis*	Ni2+	
61	3MGR	2.30	147 bp, α-satellite	*X. laevis*	Rb+	
62	3MGS	3.15	147 bp, α-satellite	*X. laevis*	Cs+	
63	3MNN	2.5	145 bp, palindromic	*X. laevis*	Ru-agent	(54)
64	3MVD	2.9	147 bp, 601	*X. laevis*	RCC1	(55)
65	3O62	3.22	146 bp, synthetic	*X. laevis*	Pt-agent	(56)
66	3REH	2.5	145 bp, α-satellite	*X. laevis*		(57)
67	3REI	2.65	145 bp, α-satellite	*X. laevis*	Pt-agent	
68	3REJ	2.55	146 bp, α-satellite	*X. laevis*		
69	3REK	2.6	146 bp, α-satellite	*X. laevis*	oxaliplatin	
70	3REL	2.7	146 bp, α-satellite	*X. laevis*	Pt-complex	
71	3TU4	3.0	147 bp, 601	*X. laevis*	Sir3 domain	(58)
72	3UT9	2.2	147 bp, 601L	*X. laevis*		(59)
73	3UTA	2.07	145 bp, α-satellite,	*X. laevis*	TTAAA	
74	3UTB	2.2	146 bp, α-satellite	*X. laevis*		
75	3W96	3.0	146 bp, palindromic	*H. sapiens*	gH2A	(60)
76	3W97	3.2	146 bp, palindromic	*H. sapiens*	gH2B	
77	3W98	3.42	146 bp, palindromic	*H. sapiens*	gH3	
78	3W99	3.0	146 bp, palindromic	*H. sapiens*	gH4	
79	3WA9	3.07	146 bp, palindromic	*H. sapiens*	H2A.Z1	(61)
80	3WAA	3.2	146 bp, palindromic	*H. sapiens*	H2A.Z2	
81	3WKJ	2.8	145 bp, palindromic	*H. sapiens*	testis H2B	(62)
82	3wtp	2.67	146 bp, palindromic	*H. sapiens*	CENP	(63)
83	3x1s	2.81	146 bp, synthetic	*H. sapiens*;		(64)
84	3x1t	2.81	146 bp, synthetic	H3, H4 *H. sapiens*; H2A, H2B *M. musculus*,	H2A, H2B testis *M. musculus*,	
85	3x1u	3.25	146 bp, synthetic	H2B, H3, H4 *H. sapiens*; H2A *M. musculus*,	H2A testis *M. musculus*,	
86	3x1v	2.92	146 bp, synthetic	H2A, H3, H4 *H. sapiens*; H2B *M. musculus*,	H2B testis *M. musculus*,	
87	4J8U	2.38	145 bp, synthetic	*X. laevis*	Os-complex	(65)
88	4J8V	2.58	145 bp, synthetic	*X. laevis*	Ru-complex	
89	4J8W	2.41	145 bp, synthetic	*X. laevis*	Os-complex	
90	4J8X	2.87	145 bp, synthetic	*X. laevis*	Ru-complex	
91	4JJN	3.09	146 bp, synthetic	*S. cerevisiae*	Sir3	(66)
92	4KGC	2.09	145 bp, synthetic	*X. laevis*	Ru-complex	(67)
93	4KUD	3.2	146 bp, synthetic	*S. cerevisiae*	Sir3 domain	(68)
94	4LD9	3.31	146 bp, 601	*X. laevis*	Sir3 domain	(69)
95	4QLC	3.2	167 bp, 601	H3, H4 *H. sapiens*; H2A, H2B *D. melanogaster*	chromatosome	(70)
96	4R8P	3.31	147 bp, 601	*X. laevis*	PRC1 domain	(71)
97	4wu8	2.45	145 bp, synthetic	*X. laevis*	Pt-intercalator	(72)

98	4wu9	2.60	145 bp, synthetic	*X. laevis*	Pt-intercalator	
99	4x23	3.5	147 bp, 601	*D. melanogaster*	CENP-C	(73)
100	4xuj	3.18	145 bp, synthetic	*X. laevis*	Ru-complex	(74)
101	4xzq	2.40	147 bp, α-satellite	*X. laevis*	H3 K115Ac	(75)
102	4ym5	4.01	144 bp, α-satellite	*H. sapiens*	Pyrimidine-pyrimidine (6-4) photoproduct	(76)
103	4ym6	3.51	145 bp, α-satellite	*H. sapiens*		
104	4ys3	3.00	147 bp, α-satellite	*X. laevis*		(75)
105	4Z5T	2.80	146 bp, α-satellite	*H. sapiens*	Testis H3.5	(77)
106	4zux	3.82	145 bp, α-satellite	*X. laevis*	Complex with SAGA DUB	(78)
107	4z66	2.50	147 bp, α-satellite	*X. laevis*		(75)
108	5AV5	2.40	147 bp, α-satellite	*H. sapiens*		(79)
109	5AV6	2.20	147 bp, α-satellite			
110	5AV8	2.20	147 bp, α-satellite			
111	5AV9	2.20	147 bp, α-satellite			
112	5AVB	2.40	147 bp, α-satellite			
113	5AVC	2.40	147 bp, α-satellite			
114	5ay8	2.80	146 bp, α-satellite	*H. sapiens*	H3.Y histone	(80)
115	5b0y	2.56	146 bp, α-satellite	*H. sapiens*	crotonylated H3 K122	(81)
116	5b0z	1.99	146 bp, α-satellite	*H. sapiens*		
117	5b1l	2.35	146 bp, α-satellite	*M. Musculus*	Mouse testis specific H3T	(82)
118	5b1m	2.34	146 bp, α-satellite	*M. Musculus*	Histones from mouse	
119	5b24	3.60	145 bp, α-satellite	*H. sapiens*	Cyclobutane pyrimidine dimer	(83)
120	5b2i	3.00	146 bp, α-satellite	*H. sapiens*		(84)
121	5b2j	2.60	146 bp, α-satellite	*H. sapiens*	Methylated DNA	
122	5b31	2.20	146 bp, α-satellite	*H. sapiens*	H2A + H2A.Z	(85)
123	5b32	2.35	146 bp, α-satellite	*H. sapiens*	H2A + H2A.Z	
124	5b33	2.93	146 bp, α-satellite	*H. sapiens*	H2A.Z	
125	5b40	3.33	146 bp, α-satellite	*H. sapiens*	Monoubiquiti-nated H2B K120 and H4 K31	(86)
126	5cp6	2.60	145 bp, synthetic	*X. laevis*	Adduct of anticancer drug	(87)
127	5CPI	2.9	146 bp, 2 satellite	*H. sapiens*		(88)
128	5CPJ	3.15	146 bp, 2 satellite	*H. sapiens*	Methylated DNA	
129	5CPK	2.6	146 bp, 2 satellite	*H. sapiens*	Methylated DNA	
130	5dnm	2.81	145 bp, synthetic	*X. laevis*	Ru(II) complex	(89)
131	5dnn	2.80	145 bp, synthetic	*X. laevis*	Ru(II) + Au(I) complexes	
132	5e5a	2.81	146 bp, α-satellite	*X. laevis*	Cytomegalovirus IE1 protein	(90)
133	5f99	2.63	147 bp MMTV-A	*X. laevis*		(91)

134	5gse	3.14	250 bp, synthetic	*H. sapiens*	Overlapping hexasome and octasome	(92)
135	5gsu	3.10	146 bp, α-satellite	*H. sapiens*	human testis-specific histone hTh2b	(93)
136	5gt0	2.82	146 bp, α-satellite	*H. sapiens*	human testis-specific histone hTh2a	
137	5gt3	2.91	146 bp, α-satellite	*H. sapiens*	human testis-specific histone hTh2b	
138	5gtc	2.70	146 bp, α-satellite	*H. sapiens*	DMAP-SH conjugate with LANA	(94)
139	5gxq	2.85	146 bp, α-satellite	*H. sapiens*	H3.6 histone variant	(95)
140	5hq2	4.50	149 bp 601	*X. laevis*	H4 K20 methyltransferase + PR/SET07	(96)
141	5jrg	2.50	145 bp, synthetic	*H. sapiens*	apyrimidinic site, tetrahydrofuran	(97)
142	5kgf	4.50	145 bp, synthetic	*X. laevis*	EM structure, P53-binding protein	(98)
143	5mlu	2.80	145 bp 601	*X. laevis*	Complex with spumavirus GAG	(99)
144	5nl0	5.40	197-bp palindromic 601L	*H. sapiens*	Complex with linker histone H1	(100)
145	5x0x	3.97	167 bp, synthetic	*X. laevis*	Complex with Snf2	(101)
146	5x0y	4.69	167 bp, synthetic	*X. laevis*	Complex with Snf2	
147	5x7x	2.18	146 bp, α-satellite	*H. sapiens*	Human H3.3	(95)

Table S3. Parameters of the DNA structure in the published NCP crystal structures. Values of radius, pitch, number of base pairs, average curvature angle per bp and average superhelix rise per bp were calculated from the double DNA helix axis path of the central 129 bp of DNA.

No	PDB code	Length, bp	Contour length, Å	Curva-ture, degrees	SH radius, Å	SH pitch, Å	bp per turn	Angle/bp step, degrees		SH rise/bp step, Å		Centre-centre, Å
								mean	min/max	mean	min/max	
1	1AOI	146	485.4	666	39.8	25.3	77.7	4.70	2.73/6.86	0.330	–0.43/1.26	4.03
2	1EQZ	146	483.3	667	39.7	25.4	77.7	4.69	2.58/6.97	0.331	–0.40/1.22	4.96
3	1F66	146	487.5	666	39.9	25.5	77.7	4.70	2.72/7.95	0.333	–0.70/1.09	7.72
4	1ID3	146	488.6	665	40.1	25.9	77.7	4.69	2.53/7.27	0.338	–0.69/1.03	3.29
5	1KX3	146	483.2	666	39.6	25.2	77. 7	4.70	2.71/6.24	0.329	–0.54/1.33	4.09
6	1KX4	146	483.2	666	39.6	26.2	78.0	4.67	2.76/7.21	0.340	–0.58/0.89	3.45
7	1KX5	147	483.9	667	39.7	24.9	78.6	4.64	2.73/6.68	0.321	–0.44/1.22	4.07
8	1M18	146	487.6	666	40.1	25.4	78.2	4.66	2.70/6.85	0.329	–0.42/1.41	8.09
9	1M19	146	487.3	667	40.1	25.3	78.2	4.67	2.80/6.47	0.328	–0.53/1.36	8.03
10	1M1A	146	486.7	664	39.9	25.6	78.2	4.66	2.12/6.64	0.331	–0.83/1.22	8.28
11	1P34	146	486.7	666	39.9	25.2	77.6	4.70	2.83/6.07	0.329	–0.59/1.29	8.35
12	1P3A	146	485.0	666	39.8	25.1	77.7	4.70	2.67/6.34	0.327	–0.45/1.44	8.23
13	1P3B	146	485.4	666	39.7	25.1	77.6	4.70	2.65/6.81	0.328	–0.52/1.50	8.08
14	1P3F	146	484.5	666	39.6	25.1	77.6	4.70	2.65/6.71	0.327	–0.64/1.40	8.21
15	1P3G	146	486.4	666	39.8	25.1	77.6	4.70	2.69/6.37	0.327	–0.49/1.27	8.21
16	1P3I	146	487.2	666	39.9	25.2	77.6	4.70	2.76/6.58	0.329	–0.46/1.34	8.31
17	1P3K	146	484.4	667	39.7	25.0	77.7	4.69	2.85/6.41	0.325	–0.35/1.33	8.17
18	1P3L	146	487.4	666	40.0	25.0	77.7	4.69	2.62/7.13	0.326	–0.46/1.25	8.26
19	1P3M	146	487.4	666	40.0	25.0	77.7	4.69	2.81/6.87	0.326	–0.40/1.13	8.24
20	1P3O	146	485.0	666	39.8	25.0	77.7	4.70	2.77/6.53	0.327	–0.47/1.38	8.26
21	1P3P	146	484.9	666	39.8	25.0	77.7	4.70	2.76/6.54	0.326	–0.39/1.34	8.24
22	1S32	146	487.2	666	40.0	24.7	77.6	4.70	2.46/7.48	0.323	–0.63/1.39	8.13
23	1U35	145	484.7	666	39.8	25.6	77.47	4.71	2.73/7.22	0.334	–0.53/1.06	7. 60
24	1ZBB	150	495.0	674	39.6	24.9	78.6	4.64	2.49/6.64	0.321	–1.04/1.25	4.41

		144	476.2	657	39.6	24.9	78.6	4.64	2.45/6.66	0.321	–0.54/1.28	5.66
25	1ZLA	146	486.8	667	39.8	25.1	77.6	4.70	2.92/7.40	0.327	–0.50/1.48	8.16
26	2CV5	146	483.7	667	39.7	26.0	77.8	4.69	2.52/6.62	0.338	–0.40/0.96	3.62
27	2F8N	145	485.3	665	39.8	25.6	77.6	4.70	2.38/7.35	0.335	–0.42/1.26	7.92
28	2FJ7	147	486.5	666	39.6	25.2	78.6	4.64	2.61/6.80	0.325	–0.75/1.40	4.15
29	2NQB	146	486.9	665	39.9	25.4	77.6	4.70	1.73/7.31	0.332	–0.57/1.35	4.26
30	2NZD	145	485.5	665	39.8	25.0	76.9	4.74	2.42/6.81	0.330	–0.46/1.09	3.86
31	2PYO	147	483.7	666	39.7	25.1	78.5	4.64	2.74/6.58	0.323	–0.41/1.21	4.14
32	3A6N	145	481.4	665	39.6	25.5	77.9	4.68	2.72/6.82	0.331	–0.55/1.19	3.60
33	3AFA	146	483.5	667	39. 8	25.7	78.0	4.68	2.31/6.39	0.334	–0.42/1.19	3.57
34	3AN2	121	407.4	580	39.6	25.9	77.9	4.68	2.72/7.51	0.337	–0.56/1.09	5.16
35	3AV1	146	484.6	667	39.9	25.6	78.0	4.68	2.34/6.42	0.333	–0.39/1.09	3.61
36	3AV2	146	485.4	667	39.9	25.8	77.9	4.68	2.31/6.58	0.335	–0.45/1.11	3.58
37	3AYW	142	475.3	657	39.9	25.5	77.9	4.68	1.49/7.64	0.332	–0.52/1.38	3.53
38	3AZE	145	483.8	664	39.8	25.4	77.8	4.68	2.19/8.79	0.331	–0.47/1.02	3.65
39	3AZF	145	484.5	666	39.8	25.6	77.9	4.68	2.86/8.66	0.333	–0.87/1.30	3.66
40	3AZG	145	484.6	665	39.9	25.5	77.9	4.68	2.73/6.62	0.332	–0.39/1.29	3.69
41	3AZH	145	485.2	664	40.0	25.7	77.9	4.68	2.48/6.66	0.334	–0.85/1.30	3.68
42	3AZI	145	486.3	666	40.0	25.5	77.8	4.69	1.87/8.32	0.333	–0.51/1.10	3.61
43	3AZJ	145	483.6	665	39.8	25.5	78.0	4.68	2.39/6.54	0.332	–0.53/1.20	3.66
44	3AZK	145	484.3	666	39.9	25.6	77.9	4.68	2.45/7.56	0.333	–0.42/1.18	3.67
45	3AZL	145	484.2	665	39.9	25.4	78.0	4.68	2.76/6.59	0.33	–0.49/1.09	3.64
46	3AZM	145	483.7	666	39.7	25.5	77.8	4.69	2.08/6.95	0.332	–0.51/1.23	3.60
47	3AZN	145	484.8	665	39.9	25.7	77.9	4.68	2.51/6.41	0.334	–0.42/1.21	3.71
48	3B6F	147	491.3	667	40.1	25.3	78.5	4.64	2.42/7.66	0.327	–0.66/1.28	4.18
49	3B6G	147	486.9	666	39.8	25.3	78.6	4.64	2.61/7.63	0.324	–1.24/1.38	4.09
50	3C1B	146	485.4	665	39.8	25.2	77.7	4.69	2.29/7.21	0.328	–0.68/1.32	4.23
51	3C1C	146	490.0	666	40.1	25.4	77.7	4.70	1.34/7.26	0.331	–0.44/1.46	4.40
52	3KUY	145	486.3	666	39.9	25.4	77.0	4.75	2.82/8.62	0.335	–0.43/1.19	3.81

53	3KWQ	146	486.8	666	40.0	25.1	77.7	4.69	2.56/7.09	0.327	–0.50/1.29	4.08
54	3KXB	146	487.0	666	40.0	25.1	77.7	4.69	2.65/7.02	0.327	–0.48/1.23	3.95
55	3LEL	147	488.5	667	40.0	25.5	78.8	4.63	2.56/6.49	0.327	–0.52/1.18	3.93
		147	489.2	666	39.9	25.2	78.7	4.63	2.58/7.36	0.324	–0.81/1.05	5.61
56	3LJA	147	486.5	667	39.9	25.0	78.5	4.64	2.80/6.52	0.322	–0.39/1.21	4.19
57	3LZ0	145	484.3	664	39.9	25. 5	77.9	4.68	2.50/6.97	0.331	–0.37/0.94	3.32
58	3LZ1	145	485.3	665	40.0	25.3	77. 9	4.68	2.55/8.29	0.329	–0.58/0.94	3.41
59	3MGP	147	490.7	672	39.7	25.4	77.8	4.69	1.92/7.05	0.331	–1.57/2.55	4.08
		147	491.3	672	39.7	25.3	77.6	4.70	1.93/7.61	0.330	–1.57/1.69	4.09
60	3MGQ	147	490.6	671	39.8	25.6	77.9	4.68	2.19/7.30	0.333	–1.59/2.06	4.14
		147	491.6	673	39.8	25.4	77.6	4.70	2.19/7.98	0.332	–1.56/1.39	4.16
61	3MGR	147	484.8	667	39.8	25.0	78.6	4.64	2.80/6.10	0.323	–0.37/1.05	4.07
62	3MGS	147	485.8	667	39.9	25.1	78.6	4.64	2.87/6.26	0.323	–0.37/1.02	4.07
63	3MNN	145	485.7	666	39.9	25.3	76.8	4.75	2.74/7.23	0.334	–0.54/1.19	3.81
64	3MVD	146	484.2	669	39.7	25.9	78.3	4.66	2.61/6.55	0.336	–0.45/1.47	3.40
65	3O62	146	495.3	673	39.8	25.1	76.4	4.77	2.08/9.67	0.333	–1.21/1.90	3.97
66	3REH	145	485.7	665	39.9	24.8	76.9	4.74	2.50/6.91	0.327	–0.49/1.14	3.85
67	3REI	145	486.2	665	39.9	24.7	76.9	4.74	2.43/6.83	0.325	–0.43/1.19	3.85
68	3REJ	146	484.1	666	39.7	25.9	78.0	4.68	2.56/6.84	0.336	–0.54/1.07	3.53
69	3REK	146	483.2	666	39.6	26.0	78.0	4.68	2.56/6.85	0.337	–0.43/1.05	3.53
70	3REL	146	483.6	669	39.7	25.9	78.0	4.68	2.40/6.74	0.336	–0.39/0.97	3.47
71	3TU4	146	487.1	669	39.8	25.3	78.0	4.68	2.85/6.12	0.329	–0.59/1.77	3.37
72	3UT9	145	483.3	665	39.8	25.6	78.0	4.67	2.55/6.81	0.332	–0.30/0.82	3.39
73	3UTA	145	483. 6	665	39.8	25.4	77.4	4.71	2.79/7.29	0.332	–0.27/1.02	3.74
74	3UTB	146	483.3	666	39.7	25.8	78.0	4.68	2.74/6.17	0.335	–0.29/0.90	3.52
75	3W96	145	483.5	666	39.8	25.7	77.9	4.68	2.67/6.75	0.334	–0.39/1.16	3.43
76	3W97	146	488.5	667	40.1	25.8	77.8	4.68	2.39/6.33	0.335	–0.59/1.05	3.42
77	3W98	145	482.6	665	39.8	25.7	78.0	4.68	2.67/6.66	0.334	–0.40/1.07	3.51
78	3W99	145	482.9	665	39.8	25.5	77.9	4.68	2.49/7.26	0.332	–0.33/1.12	3.50

79	3WA9	146	485.0	667	39.8	25.3	77.9	4.68	1.87/6.71	0.33	–0.60/1.19	3.22
80	3WAA	146	484.0	666	39. 8	25.5	77.8	4.69	0.74/6.75	0.332	–0.51/1.14	3.13
81	3WKJ	145	484.8	665	40.0	25.5	78.0	4.68	2.77/6.32	0.331	–0.45/1.13	3.39
82	3wtp	146	483.4	671	39.6	25.9	77.9	4.68	2.61/5.98	0.337	–0.36/0.95	3.51
83	3x1s	146	486.6	666	39.9	25.5	77.8	4.69	0.87/7.31	0.332	–0.90/1.71	3.50
84	3x1t	146	484.7	667	39.9	25.5	77.9	4.68	1.91/6.95	0.332	–0.45/1.22	3.33
85	3x1u	146	485.5	668	39.9	25.4	77.8	4.69	2.13/7.04	0.330	–0.39/0.99	3.41
86	3x1v	146	485.5	669	39.8	25.3	77.9	4.68	2.08/6.39	0.328	–0.32/0.96	3.45
87	4J8U	145	486.8	665	40.0	25.0	76.9	4.75	2.66/6.53	0.330	–0.43/1.11	3.87
88	4J8V	145	487.4	665	40.1	24.8	76.9	4.74	2.56/6.74	0.327	–0.42/1.09	3.87
89	4J8W	145	487.1	665	40.0	24.8	76.9	4.74	2.60/6.62	0.327	–0.48/1.01	3.89
90	4J8X	145	485.3	665	39.9	25.1	76.9	4.74	2.50/8.05	0.331	–0.41/1.17	3.83
91	4JJN	146	488.0	670	39.8	25.6	78.0	4.68	2.85/6.96	0.332	–0.43/1.11	2.81
92	4KGC	145	483.9	665	39.9	25.4	77.9	4.68	2.65/7.04	0.331	–0.37/1.04	3.35
93	4KUD	146	485.6	667	39.8	26.3	77.8	4.69	2.74/6.62	0.342	–0.37/0.85	3.48
94	4LD9	141	472.9	653	39.9	25.3	77.9	4.68	2.98/7.47	0.329	–0.44/1.08	3.63
95	4QLC	165	550.0	700	39.9	27.0	78.0	4.68	0.59/6.99	0.350	–1.07/1.17	3.54
96	4R8P	144	481.8	664	39.8	26.1	78.0	4.67	2.33/7.10	0.339	–0.33/0.88	2.96
97	4wu8	145	486.1	666	39.9	25.2	76.8	4.75	2.33/9.56	0.332	–0.58/1.08	3.75
98	4wu9	145	486.7	665	39.9	25.2	76.8	4.75	2.44/9.36	0.332	–0.49/1.12	3.81
99	4x23	146	486.9	670	39.8	25.7	78.3	4.66	2.95/6.69	0.333	–0.57/1.05	3.42
		146	487.9	671	39.8	25.8	78.3	4.66	2.85/7.00	0.334	–0.58/1.06	3.41
100	4xuj	145	484.8	665	39.8	25.0	76.9	4.75	2.42/6.93	0.329	–0.57/1.06	3.81
101	4xzq	147	485.6	667	39.9	25.0	78.6	4.64	2.88/6.70	0.322	–0.37/1.17	4.12
102	4ym5	143	486.6	665	40.3	25.6	77.1	4.73	2.45/11.18	0.337	–0.32/0.85	2.38
103	4ym6	144	488.3	667	40.3	25.8	77.2	4.73	2.65/9.40	0.339	–0.34/1.32	2.45
104	4ys3	147	487.3	667	40.0	25.0	78.6	4.64	2.88/6.53	0.322	–0.47/1.18	4.12
105	4z5t	146	485.6	668	39.9	25.8	77.8	4.69	2.29/7.53	0.335	–0.39/0.90	3.33
106	4z66	147	486.0	667	39.9	25.1	78.6	4.64	2.74/6.73	0.323	–0.32/1.13	4.17

107	4zux	145	488.8	668	40.0	26.0	77.9	4.68	2.67/7.78	0.338	–0.51/1.00	3.47
		145	488.5	668	40.0	26.2	77.9	4.68	2.79/7.14	0.34	–0.77/1.13	4.95
108	5av5	147	486.5	667	39.9	25.2	78.5	4.64	2.74/6.29	0.325	–0.48/1.02	4.03
109	5av6	147	485.4	667	39.9	25.3	78.5	4.65	2.71/6.54	0.327	–0.47/1.02	3.98
110	5av8	147	485.1	667	39.9	25.3	78.5	4.65	2.64/6.33	0.327	–0.46/1.00	4.00
111	5av9	147	485.3	667	39.9	25.3	78.5	4.65	2.70/6.72	0.326	–0.47/1.02	4.00
112	5avb	147	486.1	667	39.9	25.2	78.5	4.65	2.66/6.15	0.325	–0.44/1.02	4.02
113	5avc	147	486.8	667	40.0	25.2	78.5	4.65	2.76/6.43	0.325	–0.50/1.02	4.03
114	5ay8	145	485.0	667	39.9	25.9	78.2	4.67	2.19/6.89	0.336	–0.58/1.25	3.81
115	5b0y	146	484.2	668	39.7	25.9	77.9	4.68	2.56/6.44	0.337	–0.49/0.88	3.46
116	5b0z	146	485.1	667	39.8	25.4	77.9	4.68	2.46/6.24	0.330	–0.38/0.89	3.52
117	5b1l	144	477.9	665	39.6	25.7	77.8	4.69	2.57/6.12	0.335	–0.45/1.03	3.50
118	5b1m	146	484.9	667	39.6	25.4	77.9	4.68	2.46/5.95	0.330	–1.02/0.86	3.57
119	5b24	144	481.8	666	39.9	25.5	77.0	4.74	2.55/11.72	0.335	–0.29/0.93	2.55
120	5b2i	146	488.8	666	40.1	25.6	77.5	4.71	2.56/6.62	0.334	–0.55/0.85	4.08
121	5b2j	142	487.1	666	40.1	25.5	75.5	4.83	2.37/7.13	0.343	–0.48/0.91	4.16
122	5b31	146	483.0	667	39.8	25.6	77.9	4.68	1.55/6.68	0.333	–0.57/1.12	3.33
123	5b32	146	483.5	668	39.6	25.3	77.8	4.69	2.06/7.29	0.329	–0.46/1.00	3.35
124	5b33	146	486.4	667	39.9	25.5	77.9	4.68	1.24/7.12	0.332	–0.66/1.00	3.17
125	5b40	146	490.3	670	40.1	25.5	78.0	4.68	2.86/7.14	0.331	–0.44/0.86	3.52
126	5cp6	145	485.9	666	39.9	25.2	76.8	4.75	2.59/7.39	0.333	–0.70/1.15	3.64
127	5CPI	146	485.3	668	39.9	25.6	78.0	4.67	1.86/7.74	0.333	–0.74/1.16	3.44
128	5CPJ	146	491.7	667	40.1	25.4	78.0	4.68	0.94/6.67	0.330	–0.68/1.04	3.42
129	5CPK	145	488.0	666	40.2	26.0	77.8	4.69	2.57/7.35	0.339	–0.60/1.09	3.42
130	5dnm	145	486.1	665	40.0	25.3	76.8	4.75	2.67/7.31	0.334	–0.53/1.20	3.80
131	5dnn	145	485.6	665	39.9	25.4	76.8	4.75	2.69/7.25	0.334	–0.54/1.18	3.79
132	5e5a	146	483.3	666	39.7	25.4	78.2	4.66	2.85/6.48	0.329	–0.46/1.22	3.91
133	5f99	147	489.0	667	39.9	25.5	78.6	4.64	2.25/6.65	0.329	–0.51/1.09	3.76
134	5gse	Unusual structure										

135	5gsu	146	485.1	667	40.0	25.6	77.9	4.68	2.23/6.86	0.332	–0.31/1.10	3.23
136	5gt0	146	484.4	668	39.7	25.9	77.8	4.68	2.37/6.80	0.336	–0.40/1.09	3.46
137	5gt3	146	487.7	666	40.2	25.6	78.0	4.68	1.27/6.62	0.332	–0.38/1.13	3.37
138	5gtc	146	484.8	667	39. 9	25.5	77.9	4.68	2.13/7.08	0.332	–0.42/1.05	3.40
139	5gxq	146	490.9	667	39.9	25.5	78.0	4.68	0.29/8.13	0.331	–2.12/1.96	3.40
140	5hq2	Incomplete NCP										
141	5jrg	143	480.3	663	39.7	25.6	76.5	4.77	2.50/8.57	0.339	–0.42/1.19	3.23
142	5kgf	145	484.3	664	39.9	25.5	77.9	4.68	2.49/6.97	0.331	–0.37/0.94	3.38
143	5mlu	145	482.1	664	39.8	25.1	77.9	4.68	2.69/6.63	0.326	–0.46/0.92	3.53
144	5nl0	193	647.4	725	39.8	25.6	78.0	4.67	0.56/6.82	0.333	–0.79/0.82	3.44
145	5x0x	146	492.2	666	40.3	27.3	78.1	4.67	2.38/7.59	0.354	–1.09/1.19	3.49
146	5x0y	146	491.7	669	40.2	27.5	78.2	4.67	2.59/6.45	0.356	–0.64/1.55	3.23
147	5x7x	145	483.4	668	39.6	26.1	77.8	4.69	2.54/6.41	0.340	–0.75/0.99	3.69

Table S4. Parameters of the NCP-NCP contact in the published crystal structures.

Values that are outside typical range of NCP-NCP stacking are highlighted in bold; lower values are in plain bold, higher ones are in italic bold.

No	PDB code	Distance, Å	Axis-axis direction, degrees	NCP-NCP tilt, degrees	Tilt direction, degrees	Shift, Å	Shift angle, degrees	Rise, Å	Refe-rence
1	1AOI	67.78	179.7	13.1	11.2	41.07	–97.3	–53.92	(29)
2	1EQZ	***72.03***	179.2	***20.1***	13.0	47.44	–66.7	54.21	(30)
3	1F66	67.86	179.8	14.2	5.8	38.84	–87.3	55.64	(31)
4	1ID3	**57.05**	–180.0	11.2	177.8	**14.12**	80.0	–55.28	(32)
5	1KX3	67.39	179.6	13.0	15.2	41.12	–97.0	–53.38	
6	1KX4	66.42	–179.1	14.2	139.6	42.03	95.3	51.43	(11)
7	1KX5	67.42	179.7	12.7	12.4	40.93	–96.8	–53.58	
8	1M18	68.08	179.8	13.9	5.7	39.25	–89.2	55.63	
9	1M19	68.04	179.8	11.9	7.1	42.18	–106.1	–53.39	(33)
10	1M1A	67.82	179.9	**5.8**	32.1	38.27	–100.8	55.99	
11	1P34	68.11	179.8	15.5	5. 5	42.58	–108.5	–53.16	
12	1P3A	67.89	179.7	16.0	8.6	42.79	–107.9	–52.71	
13	1P3B	67.84	179.8	14.7	5.6	38.92	–88.4	55.57	
14	1P3F	67.85	179.8	15.3	5.0	39.01	–88.0	55.51	
15	1P3G	68.13	179.7	15.4	9.0	38.65	–87.7	56.11	
16	1P3I	68.10	179.8	14.6	4.6	39.31	–88.7	55.61	(34)
17	1P3K	67.79	179.8	15.2	5.6	42.15	–107.8	–53.09	
18	1P3L	67.90	179.7	14.7	7.7	42.53	–107.4	–52.93	
19	1P3M	67.83	179.7	15.1	7.5	42.49	–107.9	–52.88	
20	1P3O	68.01	179.6	15.9	10.1	43.08	–107.8	–52.63	
21	1P3P	67.86	179.8	15.2	6.4	42.45	–107.9	–52.95	
22	1S32	67.62	179.8	15.1	5. 7	41.93	–108.6	–53.05	(35)
23	1U35	68.17	179.9	**5.4**	59.8	42.97	–103.1	–52.92	(36)
24	1ZBB	**58.36**	**–25.4**	**6.1**	–14.8	**18.68**	68.1	55.29	(25)
25	1ZLA	67.99	179.9	15.1	1.6	42.14	–108.5	–52.92	(37)
26	2CV5	66.74	179.6	10.2	–29.1	36.72	–95.3	–53.36	(38)
27	2F8N	67.99	179.9	**4.1**	58.3	42.31	–104.2	–53.23	No ref
28	2FJ7	67.23	–179.7	**9.3**	156.1	40.90	95.0	53.37	(39)
29	2NQB	67.46	179.8	14.5	6.9	39.49	–77.0	54.70	No ref
30	2NZD	67.44	179.7	13.5	9.1	38.82	–78.9	55.15	(40)
31	2PYO	67.45	179.5	13.4	18.1	41.78	–97.7	–52.95	(41)
32	3A6N	67.42	179.6	14.6	13.8	38.62	–76.8	55.26	(42)
33	3AFA	67.39	179.5	14.8	15.0	38.46	–76.4	55.34	
34	3AN2	65.84	**0.0**	**0.0**	–175.0	36.22	91.1	53.11	(43)
35	3AV1	67.55	179.6	14.4	14.1	38.73	–77.1	55.35	(44)
36	3AV2	67.47	179.6	14.6	13.7	38.62	–76.7	55.32	

37	3AYW	67.40	179.6	15.0	13.0	38.73	–76.4	55.16	(45)
38	3AZE	67.32	179.9	**9.7**	9.9	38.94	–80.9	54.91	
39	3AZF	67.62	179.6	14.5	13.3	38.80	–77.2	55.37	
40	3AZG	67.65	179.5	14.9	14.3	38.76	–76.7	55.44	
41	3AZH	67.57	179.5	14.7	16.5	38.43	–76.8	55.57	
42	3AZI	67.42	179.9	**9.7**	8.6	39.13	–81.0	54.90	
43	3AZJ	67.43	179.4	15.1	17.1	38.29	–76.4	55.50	
44	3AZK	67.50	179.5	14.2	15.6	38.59	–77.2	55.39	
45	3AZL	67.59	179.5	14.8	14.5	38.73	–76.8	55.39	
46	3AZM	67.03	179.9	**9.3**	8.2	38.96	–81.5	54.54	
47	3AZN	67.60	179.5	14.9	15.3	38.62	–76.7	55.49	
48	3B6F	67.08	179.5	12.5	23.58	36.98	–79.9	55.96	(46)
49	3B6G	67.28	179.5	12.6	23.3	37.15	–79.8	56.10	
50	3C1B	67.56	179.6	15.9	10.0	39.17	–75.8	55.05	(47)
51	3C1C	67.86	179.8	14.8	4.4	40.12	–77.0	54.72	
52	3KUY	67.41	179.9	11.9	6.5	39.19	–80.1	54.85	(48)
53	3KWQ	67.34	179.8	13.1	6.0	39.39	–77.3	54.61	(49)
54	3KXB	67.13	179.9	13.1	5.4	39.16	–77.1	54.53	
55	3LEL	**55.80**	–178.8	**8.0**	7.3	**19.47**	–56.3	52.29	(50)
56	3LJA	67.64	179.7	12.3	11.9	38.89	–80.0	55.34	(51)
57	3LZ0	67.40	–179.8	**7.7**	155.1	37.83	83.9	–55.78	(52)
58	3LZ1	67.39	–179.8	**7.7**	156.1	37.89	83.8	–55.73	
59	3MGP	67.71	179.2	17.3	17.9	38.53	–72.8	55.68	(53)
60	3MGQ	67.56	179.3	16.5	18.2	38.44	–73.4	55.55	
61	3MGR	67.57	179.7	12.2	12.5	38.77	–80.2	55.35	
62	3MGS	67.58	179.7	12.1	12.1	38.83	–80.3	55.31	
63	3MNN	67.57	179.9	11.9	4.2	39.48	–80.3	54.83	(54)
64	3MVD	***100.27***	160.4	***23.2***	79.8	***93.01***	–123.5	–35.41	(55)
		102.68	0.0	**0.0**	92.6	**21.12**	92.5	–100.49	
65	3O62	67.33	179.8	10.3	11.0	38.76	–79.9	55.06	(56)
66	3REH	67.53	179.7	13.5	10.6	38.66	–78.9	55.37	(57)
67	3REI	67.52	179.7	13.1	11.8	38.61	–79.2	55.39	
68	3REJ	66.48	–179.2	13.5	144.7	33.29	82.5	–57.54	
69	3REK	66.41	–179.3	12.9	145.3	33.30	82.6	–57.46	
70	3REL	66.55	–179.2	13.5	143.6	33.09	82.3	–57.73	
71	3TU4	***95.84***	**58.2**	14.9	61.8	**1.79**	–157.8	95.83	(58)
72	3UT9	67.43	179.9	**8.0**	15.7	38.53	–83.8	55.34	(59)
73	3UTA	67.66	179.7	12.6	11.9	38.77	–80.3	55.45	
74	3UTB	66.35	–179.2	13.1	134.2	33.33	82.6	–57.37	
75	3W96	67.38	179.9	9.2	10.4	39.06	–80.9	54.90	(60)
76	3W97	67.89	179.9	**9.4**	9.2	39.73	–80.9	55.05	
77	3W98	67.42	179.9	**9.8**	9.2	39.20	–80.5	54.86	
78	3W99	67.36	179.8	10.2	14.6	38.70	–79.8	55.13	
79	3WA9	67.06	179.4	14.8	18.1	37.73	–75.2	55.44	(61)
80	3WAA	67.35	179.4	14.8	17.6	37.97	–75.2	55.62	

81	3WKJ	67.75	179.5	13.3	19.9	38.11	–79.0	56.02	(62)
82	3wtp	66.26	–179.7	10.6	–20.9	40.77	–81.8	52.23	(63)
83	3x1s	67.37	179.8	10.5	14.2	38.66	–80.1	55.18	(64)
84	3x1t	67.39	179.5	14.4	14.6	38.54	–76.0	55.29	
85	3x1u	67.22	179.9	9.8	10.2	38.90	–80.2	54.82	
86	3x1v	67.04	179.5	12.8	20.7	37.88	–79.2	55.31	
87	4J8U	67.61	179.7	12.6	12.2	38.72	–79.5	55.43	(65)
88	4J8V	67.62	179.6	12.9	14.2	38.58	–79.4	55.54	
89	4J8W	67.58	179.7	12.8	13.0	38.71	–79.5	55.40	
90	4J8X	67.56	179.7	12.8	14.5	38.46	–79.5	55.55	
91	4JJN	***95.86***	**58.4**	13.7	64.0	**2.06**	–117.8	95.82	(66)
92	4KGC	67.58	179.5	12.9	21.9	37.75	–79.5	56.05	(67)
93	4KUD	***93.72***	**–57.7**	***23.6***	153.1	**20.16**	3.3	–91.52	(68)
94	4LD9	***93.22***	**–57.3**	***27.0***	163.2	**22.49**	2.8	–90.47	(69)
95	4QLC	97.56	121.6	78.9	–142.5	97.36	9.4	–6.35	(70)
		77.23	166.9	85.5	–102.2	10.94	–60.8	–76.46	
96	4R8P	***101.17***	**74.9**	2.9	–142.6	30.19	132.1	–96.56	(71)
97	4wu8	67.43	179.8	11.8	9.1	38.93	–80.1	55.05	(72)
98	4wu9	67.17	179.9	9.8	5.8	38.95	–81.8	54.72	
99	4x23	**54,16**	173.1	**0.5**	109.0	**16.39**	–4.6	51.62	(73)
		**59,29**	**–7.8**	15.9	17.2	**20.16**	–67.6	–55.76	
100	4xuj	67.56	179.7	12.5	13.6	38.62	–79.5	55.43	(74)
101	4xzq	67.34	179.7	12.3	13.6	40.78	–96.5	–53.59	(75)
102	4ym5	67.10	179.4	12.1	32.5	36.35	–79.2	56.40	(76)
103	4ym6	66.95	179.1	7.3	125.8	35.68	–92.6	56.65	
104	4ys3	67.40	179.7	12.3	12.7	40.72	–96.2	–53.70	(75)
105	4Z5T	67.17	–180.0	**4.8**	177.1	39.07	87.8	–54.63	(77)
106	4z66	67.42	179.7	12.7	12.8	40.86	–96.8	–53.63	(75)
107	4zux	***95.46***	180.0	23.9	–90.1	37.48	–26.9	87.80	(78)
		98.21	**–92.3**	80.2	171.2	91.86	–106.5	34.74	
108	5AV5	67.69	179.6	12.8	15.5	38.42	–79.4	55.73	(79)
109	5AV6	67.71	179.8	11.8	11.7	38.84	–80.3	55.46	
110	5AV8	67.63	179.7	11.9	12.4	38.70	–80.2	55.46	
111	5AV9	67.60	179.7	11.9	12.0	38.77	–80.2	55.37	
112	5AVB	67.66	179.6	13.0	15.7	38.31	–79.2	55.77	
113	5AVC	67.78	179.6	12.8	14.8	38.58	–79.5	55.73	
114	5ay8	**54.18**	180.0	13.5	0.4	**21.64**	**–49.6**	**49.67**	(80)
115	5b0y	67.00	–179.9	**9.2**	–11.8	40.38	–82.0	53.46	(81)
116	5b0z	66.84	179.2	13.9	30.6	36.49	–79.0	56.00	
117	5b1l	66.74	–179.9	9.03	–10.7	40.68	–83.1	52.91	(82)
118	5b1m	66.85	179.2	14.0	30.1	36.90	–79.2	55.74	
119	5b24	67.07	179.7	8.8	25.7	37.59	–83.7	55.55	(83)
120	5b2i	67.38	179.8	8.7	15.2	38.72	–83.7	55.14	(84)
121	5b2j	67.48	179.9	11.2	4.1	39.40	–81.3	54.79	
122	5b31	67.31	179.5	14.7	16.7	38.10	–75.6	55.49	(85)

123	5b32	66.17	–179.9	10.1	–5.3	40.60	–81.2	52.25	
124	5b33	67.21	179.4	15.4	17.0	37.80	–74.5	55.57	
125	5b40	**62.07**	**120.4**	**19.3**	56.0	**11.14**	–106.5	–61.06	(86)
126	5cp6	67.29	179.7	11.6	14.3	38.26	–80.0	55.35	(87)
127	5CPI	67.30	179.6	12.2	18.2	38.13	–78.6	55.46	(88)
128	5CPJ	66.80	179.4	13.2	27.7	36.41<br>9	–78.1	56.00	
129	5CPK	66.82	179.5	10.9	35.6	41.55	–94.7	–52.33	
130	5dnm	67.59	179.6	12.5	17.3	38.21	–79.8	55.75	(89)
131	5dnn	67.65	179.6	12.6	18.5	38.10	–79.7	55.91	
132	5e5a	67.53	179.9	13.2	3.9	39.93	–78.2	54.46	(90)
133	5f99	67.73	179.9	**7.6**	13.7	39.57	–83.3	54.97	(91)
134	5gse	Unusual structure							(92)
135	5gsu	67.69	179.3	15.7	19.2	38.40	–74.7	55.74	(93)
136	5gt0	67.08	–179.9	9.5	–7.8	38. 60	–94.3	–54.87	
137	5gt3	67.85	179.3	15.3	20.1	38.29	–75.3	56.01	
138	5gtc	67.32	179.9	9.3	5.7	39.49	–81.3	54.52	(94)
139	5gxq	67.53	179.4	15.1	18.4	38.13	–75.8	55.73	(95)
140	5hq2	No stacking. Incomplete NCP							(96)
141	5jrg	66.62	–179.9	4.2	155.8	37.60	–89.8	55.00	(97)
142	5kgf	Cryo-EM. Single molecule							(98)
143	5mlu	67.24	–179.7	8.7	141.5	36.63	84.2	–56.39	(99)
144	5nl0	**61.73**	**0.0**	**0.0**	–69.0	21.55	92.7	57.84	(100)
145	5x0x	Cryo-EM. Single molecule							(101)
146	5x0y	Cryo-EM. Single molecule							
147	5x7x	66.39	–179.8	8.4	–19.6	40.54	–83.1	52.57	(95)

Table S5. Summary of availability, location and participation of the histone tails in NCP-NCP stacking from the literature data reporting NCP structures. This table take into account H4 and H2A considering that these full-length tails contain respectively 23 and 20 amino acids.

No	PDB code	NCP1				NCP2			
		H2A		H4		H2A		H4	
		a.a. (chain)	Contacts	a.a. (chain)	Contacts	a.a. (chain)	Contacts	a.a. (chain)	Contacts
1	1AOI	**4-20 (C)**	**DNA1+DNA2**	**20-23 (B)**	**DNA1**	**12-20 (G)**	**DNA2**	**16-23 (B)**	**AcIsl1**
2	1EQZ	2-20 (E)	DNA1	8-23 (H)	AcIsl2+DNA1	1-20 (A)	DNA1+DNA2	14-23 (D)	DNA2
3	1F66	16-20 (G)	DNA1	17-23 (F)	AcIsl2+DNA1	16-20 (C)	DNA2	23 (B)	DNA2
4	1ID3	16-20 (C)	DNA1	absent	--	13-18 (G)	DNA2	18-23 (F)	DNA2?
5	1KX3	14-20 (C)	DNA1+DNA2	21-23 (B)	DNA1	14-20 (G)	DNA2	**16-23** (F)	AcIsl1
6	1KX4	14-20 (G)	DNA1	absent	--	16-20 (C)	DNA2	20-23 (B)	AcIsl1
**7**	1KX5	**15-20 (C)**	**DNA1**	**15-23 (B)**	**DNA1**	**13-20 (G)**	**DNA2**	**16-23 (F)**	**AcIsl1+DNA2**
8	1M18	14-20 (G)	DNA1	20-23 (F)	AcIsl2	14-20 (C)	DNA2	absent	--
9	1M19	14-20 (C)	DNA1+DNA2	absent	--	15-20 (G)	DNA2	17-23 (F)	AcIsl1+DNA2
10	1M1A	14-20 (G)	DNA1	10-23 (F)	AcIsl2	14-20 (C)	DNA2	23 (B)	DNA2
11	1P34	12-20 (C)	DNA1+DNA2	20-23 (B)	DNA1	13-20 (G)	DNA2	22-23 (F)	DNA2
12	1P3A	14-20 (C)	DNA1+DNA2	absent	--	16-20 (G)	DNA2	21-23 (F)	AcIsl1
13	1P3B	15-20 (G)	DNA1	22-23 (F)	AcIsl2	13-20 (G)	DNA2	absent	--
14	1P3F	13-20 (G)	DNA1	21-23 (F)	AcIsl1	15-20 (C)	DNA1+DNA2	23 (B)	DNA2
15	1P3G	13-20 (G)	DNA1	**16-23** (F)	AcIsl2+DNA1	14-20 (C)	DNA1+DNA2	absent	--
16	1P3I	16-20 (G)	DNA1	absent	--	15-20 (C)	DNA2	absent	--
17	1P3K	15-20 (C)	DNA1+DNA2	absent	--	14-20 (G)	DNA2	22-23 (F)	AcIsl1
18	1P3L	15-20 (C)	DNA1+DNA2	absent	--	11-20 (G)	DNA2	20-23 (F)	AcIsl1
19	1P3M	14-20 (C)	DNA1+DNA2	absent	--	15-20 (G)	DNA2	21-23 (F)	AcIsl1
20	1P3O	14-20 (C)	DNA1+DNA2	21-23 (B)	DNA1	14-20 (G)	DNA2	absent	--
21	1P3P	14-20 (C)	DNA1+DNA2	22-23 (B)	DNA1	12-20 (G)	DNA2	21-23 (F)	AcIsl1
22	1S32	12-20 (C)	DNA1+DNA2	22-23 (B)	DNA1	13-20 (G)	DNA2	**15-23** (F)	AcIsl1+DNA2
23	1U35	14-20 (C)	DNA1	absent	--	14-20 (G)	DNA2	20-23 (F)	AcIsl1+DNA2
**24**	1ZBB	**13-20 (G)**	**DNA1**	**16-23 (F)**	**DNA1+DNA2**	**13-20 (g)**	**DNA2**	**16-23 (f)**	**DNA1+DNA2**
25	1ZLA	14-20 (C)	DNA1	absent	--	14-20 (G)	DNA2	19-23 (F)	AcIsl1+DNA2

26	2CV5	11-20 (C)	DNA1	absent	--	15-20 (G)	DNA2	18-23 (F)	AcIsl1+DNA2
27	2F8N	absent	--	absent	--	absent	--	19-23 (F)	AcIsl1
28	2FJ7	14-20 (C)	DNA1	absent	--	14-20 (G)	DNA2	19-23 (F)	AcIsl1
29	2NQB	14-20 (G)	DNA1	17-23 (F)	AcIsl1	14-20 (C)	DNA1+DNA2	22-23 (B)	DNA2
30	2NZD	14-20 (G)	DNA1	**16-23** (F)	AcIsl1	14-20 (C)	DNA1+DNA2	21-23 (B)	DNA2
31	2PYO	13-20 (C)	DNA1+DNA2	absent	--	11-20 (G)	DNA2	15-23 (F)	AcIsl1+DNA2
32	3A6N	15-20 (G)	DNA1	19-23 (F)	AcIsl1	14-20 (C)	DNA2	22-23 (B)	DNA2
33	3AFA	15-20 (G)	DNA1	19-23 (F)	AcIsl1	11-20 (C)	DNA1+DNA2	absent	--
34	3AN2	16-20 (G)	DNA1	absent	--	17-21 (C)	DNA2	absent	--
35	3AV1	15-20 (G)	DNA1	19-23 (F)	AcIsl2+DNA1	14-20 (C)	DNA1+DNA2	absent	--
36	3AV2	15-20 (G)	DNA1	19-23 (F)	AcIsl2+DNA1	14-20 (C)	DNA1+DNA2	absent	--
37	3AYW	16-20 (G)	DNA1	19-23 (F)	AcIsl2+DNA1	11-20 (C)	DNA1+DNA2	absent	--
38	3AZE	15-20 (G)	DNA1	18-23 (F)	AcIsl2+DNA1	11-20 (C)	DNA2	absent	--
39	3AZF	15-20 (G)	DNA1	19-23 (F)	AcIsl2+DNA1	11-20 (C)	DNA1+DNA2	absent	--
40	3AZG	15-20 (G)	DNA1	19-23 (F)	AcIsl2+DNA1	11-20 (C)	DNA1+DNA2	absent	--
41	3AZH	15-20 (G)	DNA1	18-23 (F)	AcIsl2+DNA1	13-20 (C)	DNA1+DNA2	absent	--
42	3AZI	15-20 (G)	DNA1	19-23 (F)	AcIsl2+DNA1	11-20 (C)	DNA2	absent	--
43	3AZJ	15-20 (G)	DNA1	19-23 (F)	AcIsl2	11-20 (C)	DNA2	absent	--
44	3AZK	14-20 (G)	DNA1	19-23 (F)	AcIsl2+DNA1	11-20 (C)	DNA2	absent	--
45	3AZL	14-20 (G)	DNA1	17-23 (F)	AcIsl2+DNA1	11-20 (C)	DNA1+DNA2	absent	--
46	3AZM	15-20 (G)	DNA1	19-23 (F)	AcIsl2+DNA1	13-20 (C)	DNA2	absent	--
47	3AZN	15-20 (G)	DNA1	19-23 (F)	AcIsl2+DNA1	11-20 (C)	DNA2	absent	--
48	3B6F	13-20 (G)	DNA1	**16-23** (F)	AcIsl2+DNA1	15-20 (C)	DNA2	absent	--
49	3B6G	13-20 (G)	DNA1	**16-23** (F)	AcIsl2+DNA1	15-20 (C)	DNA2	absent	--
50	3C1B	16-20 (G)	DNA1	**16-23** (F)	AcIsl2+DNA1	14-20 (C)	DNA2	23 (B)	DNA2
51	3C1C	16-20 (G)	DNA1	19-23 (F)	AcIsl2	14-20 (C)	DNA2	absent	--
52	3KUY	14-20 (G)	DNA1	**16-23** (F)	AcIsl2	14-20 (C)	DNA2	21-23 (B)	DNA2
53	3KWQ	14-20 (G)	DNA1	20-23 (F)	AcIsl2	14-20 (C)	DNA2	absent	--
54	3KXB	20 (G)	DNA1	**16-23** (F)	AcIsl2	14-20 (C)	DNA2	absent	--
**55**	3LEL	**13-20 (G)**	**DNA1**	**17-23 (F)**	**AcIsl2+DNA1**	**15-20 (M)**	**DNA2**	**absent**	**--**
56	3LJA	13-20 (G)	DNA1	**16-23** (F)	AcIsl2+DNA1	15-20 (C)	DNA2	absent	--

57	3LZ0	16-20 (C)	DNA1	20-23 (B)	AcIsl2	14-20 (G)	DNA2	absent	--
58	3LZ1	16-20 (C)	DNA1	20-23 (B)	AcIsl2	14-20 (G)	DNA2	absent	--
59	3MGP	14-20 (G)	DNA1	**16-23** (F)	AcIsl2+DNA1	15-20 (C)	DNA2	absent	--
60	3MGQ	13-20 (G)	DNA1	**16-23** (F)	AcIsl2+DNA1	15-20 (C)	DNA2	absent	--
61	3MGR	13-20 (G)	DNA1	**16-23** (F)	AcIsl2+DNA1	15-20 (C)	DNA2	absent	--
62	3MGS	13-20 (G)	DNA1	**16-23** (F)	AcIsl2+DNA1	15-20 (C)	DNA2	absent	--
63	3MNN	14-20 (G)	DNA1	**16-23** (F)	AcIsl2	14-20 (C)	DNA2	21-23 (B)	DNA2
64	3MVD	NCP + protein. No stacking							
65	3O62	14-20 (G)	DNA1	18-23 (F)	AcIsl2+DNA1	14-20 (C)	DNA2	23 (B)	--
66	3REH	14-20 (G)	DNA1	**16-23** (F)	AcIsl2	14-20 (C)	DNA1+DNA2	21-23 (B)	DNA2
67	3REI	14-20 (G)	DNA1	**16-23** (F)	AcIsl2	14-20 (C)	DNA1+DNA2	21-23 (B)	DNA2
68	3REJ	16-20 (C)	DNA1	20-23 (B)	AcIsl2	14-20 (G)	DNA2	absent	--
69	3REK	16-20 (C)	DNA1	20-23 (B)	AcIsl2	14-20 (G)	DNA2	absent	--
70	3REL	16-20 (C)	DNA1	20-23 (B)	AcIsl2	14-20 (G)	DNA2	absent	--
71	3TU4	NCP + protein. No stacking							
72	3UT9	16-20 (G)	DNA1	**16-23** (F)	AcIsl2	14-20 (C)	DNA2	absent	--
73	3UTA	14-20 (G)	DNA1	**16-23** (F)	AcIsl2+DNA1	14-20 (C)	DNA2	21-23 (B)	DNA2
74	3UTB	16-20 (C)	DNA1	20-23 (B)	AcIsl2	14-20 (G)	DNA2	absent	--
75	3W96	16-20 (G)	DNA1	20-23 (F)	AcIsl2	12-20 (C)	DNA2	absent	--
76	3W97	16-20 (G)	DNA1	19-23 (F)	AcIsl2	13-20 (C)	DNA2	absent	--
77	3W98	15-20 (G)	DNA1	19-23 (F)	AcIsl2	13-20 (C)	DNA2	absent	--
78	3W99	17-20 (G)	DNA1	19-23 (F)	AcIsl2+DNA1	14-20 (C)	DNA2	absent	--
79	3WA9	17-20 (G)	DNA1	19-23 (F)	AcIsl2+DNA1	15-20 (C)	DNA2	absent	--
80	3WAA	17-20 (G)	DNA1	19-23 (F)	AcIsl2+DNA1	14-20 (C)	DNA2	absent	--
81	3WKJ	15-20 (G)	DNA1	19-23 (F)	AcIsl2+DNA1	12-20 (C)	DNA2	absent	--
82	3wtp	14-20 (G)	DNA1	18-23 (F)	AcIsl2+DNA1	12-20 (C)	DNA2	absent	--
83	3x1s	15-20 (G)	DNA1	19-23 (F)	AcIsl2	14-20 (C)	DNA2	absent	--
84	3x1t	14-20 (G)	DNA1	**16-23 (F)**	**AcIsl2+DNA1**	14-20 (C)	DNA2	22-23 (B)	DNA2
85	3x1u	11-20 (G)	DNA1	**16-23 (F)**	**AcIsl2+DNA1**	14-20 (C)	DNA2	20-23 (B)	Other NCP
86	3x1v	11-20 (G)	DNA1	**16-23 (F)**	**AcIsl2**	13-20 (C)	DNA2	absent	--
87	4J8U	14-20 (G)	DNA1	**16-23 (F)**	**AcIsl2+ AcIsl2**	14-20 (C)	DNA2+DNA1	21-23 (B)	DNA2

88	4J8V	14-20 (G)	DNA1	**16-23 (F)**	**AcIsl2+ AcIsl2**	14-20 (C)	DNA2	21-23 (B)	DNA2
89	4J8W	14-20 (G)	DNA1	**16-23 (F)**	**AcIsl2**	14-20 (C)	DNA2	21-23 (B)	DNA2
90	4J8X	14-20 (G)	DNA1	**16-23 (F)**	**AcIsl2**	14-20 (C)	DNA2	21-23 (B)	DNA2
91	4JJN	NCP + protein. No stacking							
92	4KGC	14-20 (G)	DNA1	**16-23 (F)**	**AcIsl2**	14-20 (C)	DNA2	21-23 (B)	DNA2
93	4KUD	NCP + protein. No stacking							
94	4LD9	NCP + protein. No stacking							
95	4QLC	NCP + protein. No stacking							
96	4R8P	NCP + protein. No stacking							
97	4wu8	14-20 (G)	DNA1	**16-23 (F)**	**AcIsl2**	14-20 (C)	DNA2	21-23 (B)	DNA2
98	4wu9	14-20 (G)	DNA1	**16-23 (F)**	**AcIsl2**	14-20 (C)	DNA2	21-23 (B)	DNA2
99	4x23	15-20 (G)	DNA1	absent	--	15-20 (C)	DNA2	absent	--
100	4xuj	14-20 (G)	DNA1	**16-23 (F)**	AcIsl2+DNA1	14-20 (C)	DNA2+ DNA1	21-23 (B)	DNA2
101	4xzq	14-20 (G)	DNA1	absent	--	14-20 (C)	DNA2+ DNA1	absent	--
102	4ym5	16-20 (G)	DNA1	**16-23 (F)**	**AcIsl2**	11-20 (C)	DNA2	absent	--
103	4ym6	15-20 (G)	DNA1	19-23 (F)	AcIsl2+DNA1	11-20 (C)	DNA2	absent	--
104	4ys3	14-20 (G)	DNA1	absent	--	14-20 (C)	DNA2	absent	--
105	4Z5T	14-20 (C)	DNA1	20-23 (B)	AcIsl2+DNA1	15-20 (G)	DNA2	absent	--
106	4zux	NCP + protein. No stacking							
107	4z66	14-20 (G)	DNA1	21-23 (F)	AcIsl2	14-20 (C)	DNA2+ DNA1	absent	--
108	5AV5	14-20 (G)	DNA1	17-23 (F)	AcIsl2+DNA1	11-20 (C)	DNA2+ DNA1	21-23 (B)	DNA2
109	5AV6	14-20 (G)	DNA1	17-23 (F)	AcIsl2+DNA1	13-20 (C)	DNA2+ DNA1	21-23 (B)	DNA2
110	5AV8	14-20 (G)	DNA1	17-23 (F)	AcIsl2+DNA1	13-20 (C)	DNA2	21-23 (B)	DNA2
111	5AV9	14-20 (G)	DNA1	17-23 (F)	AcIsl2+DNA1	13-20 (C)	DNA2	21-23 (B)	DNA2
112	5AVB	14-20 (G)	DNA1	**16-23 (F)**	AcIsl2+DNA1	13-20 (C)	DNA2+ DNA1	21-23 (B)	DNA2
113	5AVC	14-20 (G)	DNA1	**16-23 (F)**	AcIsl2+DNA1	13-20 (C)	DNA2+ DNA1	21-23 (B)	DNA2
114	5ay8	14-20 (C)	DNA1	absent	--	15-20 (G)	DNA2	absent	--
115	5b0y	15-20 (G)	DNA1	19-23 (F)	AcIsl2	14-20 (C)	DNA2	absent	--
116	5b0z	15-20 (G)	DNA1	20-23 (F)	AcIsl2	11-20 (C)	DNA2+ DNA1	absent	--
117	5b1l	15-20 (G)	DNA1	19-23 (F)	AcIsl2+DNA1	11-20 (C)	DNA2	absent	--
118	5b1m	15-20 (G)	DNA1	19-23 (F)	AcIsl2	11-20 (C)	DNA2+ DNA1	absent	--

119	5b24	15-20 (G)	DNA1	19-23 (F)	AcIsl2+DNA1	11-20 (C)	DNA2	absent	--
120	5b2i	14-20 (G)	DNA1	18-23 (F)	AcIsl2+DNA1	13-20 (C)	DNA2+ DNA1	21-23 (B)	Other NCP
121	5b2j	14-20 (G)	DNA1	18-23 (F)	AcIsl2+DNA1	13-20 (C)	DNA2	21-23 (B)	DNA2
122	5b31	16-20 (G)	DNA1	19-23 (F)	AcIsl2	13-20 (C)	DNA2	absent	--
123	5b32	16-20 (G)	DNA1	19-23 (F)	AcIsl2+DNA1	11-22 (C)	DNA2	absent	--
124	5b33	17-20 (G)	DNA1	19-23 (F)	AcIsl2+DNA1	16-22 (C)	DNA2	absent	--
125	5b40	15-20 (G)	DNA1	22-23 (F)	No contact	14-20 (C)	DNA2	absent	--
126	5cp6	15-20 (G)	DNA1	**16-23 (F)**	AcIsl2+DNA1	14-20 (C)	DNA2	21-23 (B)	DNA2
127	5CPI	15-20 (G)	DNA1	19-23 (F)	AcIsl2+DNA1	11-20 (C)	DNA2	absent	--
128	5CPJ	15-20 (G)	DNA1	19-23 (F)	AcIsl2+DNA1	15-20 (C)	DNA2	absent	--
129	5CPK	15-20 (G)	DNA1	19-23 (F)	AcIsl2+DNA1	14-20 (C)	DNA2	absent	--
130	5dnm	14-20 (G)	DNA1	**16-23 (F)**	AcIsl2+DNA1	14-20 (C)	DNA2+ DNA1	21-23 (B)	Other NCP
131	5dnn	14-20 (G)	DNA1	**16-23 (F)**	AcIsl2+DNA1	14-20 (C)	DNA2	21-23 (B)	DNA2
132	5e5a	14-20 (G)	DNA1	17-23 (F)	AcIsl2+DNA1	14-20 (C)	DNA2	absent	--
133	5f99	9-20 (G)	DNA1	**11-23 (F)**	AcIsl2+DNA1	11-20 (C)	DNA2	20-23 (B)	Other NCP
134	5gse	Unusual structure							
135	5gsu	16-20 (G)	DNA1	17-23 (F)	AcIsl2	15-20 (C)	DNA2	absent	--
136	5gt0	14-20 (C)	DNA1	absent	--	13-20 (G)	DNA2	17-23 (F)	AcIsl1
137	5gt3	16-20 (G)	DNA1	18-23 (F)	AcIsl2	13-20 (C)	DNA2	21-23 (B)	--
138	5gtc	12-20 (G)	DNA1	17-23 (F)	AcIsl2+DNA1	11-20 (C)	DNA2	absent	--
139	5gxq	15-20 (G)	DNA1	19-23 (F)	AcIsl2+DNA1	11-20 (C)	DNA2	absent	--
140	5hq2	No stacking. Incomplete NCP							
141	5jrg	10-20 (G)	DNA1	17-23 (F)	AcIsl2+DNA1	12-20 (C)	DNA2	absent	--
142	5kgf	Cryo-EM. Single molecule							
143	5mlu	16-20 (C)	DNA1	19-23 (B)	AcIsl2	14-20 (G)	DNA2	absent	--
144	5nl0	16-20 (G)	DNA1	20-23 (F)	DNA2	14-20 (C)	DNA2	20-23 (B)	DNA1
145	5x0x	Cryo-EM. Single molecule							
146	5x0y	Cryo-EM. Single molecule							
147	5x7x	14-20 (G)	DNA1	17-23 (F)	AcIsl2+DNA1	11-20 (C)	DNA2	absent	--

Supporting references

1. Lavery, R., Moakher, M., Maddocks, J.H., Petkeviciute, D. and Zakrzewska, K. (2009) Conformational analysis of nucleic acids revisited: Curves+. *Nucleic Acids Res.*, **37**, 5917-5929.
2. Blanchet, C., Pasi, M., Zakrzewska, K. and Lavery, R. (2011) CURVES+ web server for analyzing and visualizing the helical, backbone and groove parameters of nucleic acid structures. *Nucleic Acids Res.*, **39**, W68-W73.
3. Fan, Y., Korolev, N., Lyubartsev, A.P. and Nordenskiöld, L. (2013) An advanced coarse-grained nucleosome core particle model for computer simulations of nucleosome-nucleosome interactions under varying ionic conditions. *PLoS One*, **8**, e54228.
4. Lyubartsev, A.P., Korolev, N., Fan, Y. and Nordenskiöld, L. (2015) Multiscale modelling of nucleosome core particle aggregation. *J. Phys. Condens. Matter*, **27**, 064111.
5. Lyubartsev, A.P. and Nordenskiöld, L. (1997) Monte Carlo simulation study of DNA polyelectrolyte properties in the presence of multivalent polyamine ions. *J.Phys.Chem.B*, **101**, 4335-4342.
6. Korolev, N., Lyubartsev, A.P., Rupprecht, A. and Nordenskiöld, L. (1999) Experimental and Monte Carlo simulation studies on the competitive binding of Li^+, Na^+, and K^+ ions to DNA in oriented DNA fibers. *J.Phys.Chem.B*, **103**, 9008-9019.
7. Korolev, N., Lyubartsev, A.P., Rupprecht, A. and Nordenskiöld, L. (1999) Competitive binding of Mg^{2+}, Ca^{2+}, Na^+, and K^+ to DNA in oriented DNA fibers: experimental and Monte Carlo simulation results. *Biophys.J.*, **77**, 2736-2749.
8. Korolev, N., Lyubartsev, A.P., Rupprecht, A. and Nordenskiöld, L. (2001) Competitive substitution of hexammine cobalt(III) for Na^+ and K^+ ions in oriented DNA fibers. *Biopolymers*, **58**, 268-278.
9. Montoro, J.C.G. and Abascal, J.L.F. (1995) Ionic distribution around simple DNA models. I. Cylindrically averaged properties. *J.Chem.Phys.*, **103**, 8273-8284.
10. Montoro, J.C.G. and Abascal, J.L.F. (1998) Ionic distribution around simple B-DNA models. II. Deviations from cylindrical symmetry. *J.Chem.Phys.*, **109**, 6200-6210.
11. Davey, C.A., Sargent, D.F., Luger, K., Maeder, A.W. and Richmond, T.J. (2002) Solvent mediated interactions in the structure of nucleosome core particle at 1.9 Å resolution. *J.Mol.Biol.*, **319**, 1097-1113.
12. Haliloglu, T., Bahar, I. and Erman, B. (1997) Gaussian dynamics of folded proteins. *Phys. Rev. Lett.*, **79**, 3090-3093.
13. Biswas, M., Voltz, K., Smith, J.C. and Langowski, J. (2011) Role of histone tails in structural stability of the nucleosome. *PLoS Comput. Biol.*, **7**, e1002279.
14. Voltz, K., Trylska, J., Tozzini, V., Kurkal-Siebert, V., Langowski, J. and Smith, J. (2008) Coarse-grained force field for the nucleosome from self-consistent multiscaling. *J.Comp.Chem.*, **29**, 1429-1439.
15. Korolev, N., Yu, H., Lyubartsev, A.P. and Nordenskiöld, L. (2014) Molecular Dynamics simulations demonstrate the regulation of DNA-DNA attraction by H4 histone tail acetylations and mutations. *Biopolymers*, **101**, 1051-1064.
16. Nordenskiöld, L., Lyubartsev, A.P. and Korolev, N. (2008) DNA-DNA interaction. In Dias, R. S. and Lindman, B. (eds.), *DNA Interactions with Polymers and Surfactants*. John Wiley & Sons, Inc., London, pp. 209-237.
17. Korolev, N. and Nordenskiöld, L. (2007) H4 histone tail mediated DNA-DNA interaction and effects on DNA structure, flexibility and counterion binding. A molecular dynamics study. *Biopolymers*, **86**, 409–423.

18. Limbach, H.J., Arnold, A., Mann, B.A. and Holm, C. (2006) ESPResSo - an extensible simulation package for research on soft matter systems. *Comp.Phys.Comm.*, **174**, 704-727.
19. Korolev, N., Allahverdi, A., Yang, Y., Fan, Y., Lyubartsev, A.P. and Nordenskiöld, L. (2010) Electrostatic origin of salt-induced nucleosome array compaction. *Biophys. J.*, **99** 1896-1905.
20. Yang, Y., Lyubartsev, A.P., Korolev, N. and Nordenskiöld, L. (2009) Computer modeling reveals that modifications of the histone tail charges define salt-dependent interaction of the nucleosome core particles. *Biophys.J.*, **96**, 2082-2094.
21. Deserno, M. and Holm, C. (1998) How to mesh up Ewald sums. I. A theoretical and numerical comparison of various particle mesh routines. *J.Chem.Phys.*, **109**, 7678-7693.
22. Cheatham, T.E., III and Kollman, P.A. (1997) Molecular dynamics simulations highlight the structural differences among DNA:DNA, RNA:RNA, and DNA:RNA structures. *J.Amer.Chem.Soc.*, **119**, 4805-4825.
23. Lyubartsev, A.P. and Laaksonen, A. (1998) Molecular dynamics simulations of DNA in solution with different counter-ions. *J.Biomol.Struct.Dyn.*, **16**, 579-592.
24. Korolev, N., Lyubartsev, A.P., Nordenskiöld, L. and Laaksonen, A. (2001) Spermine: an "invisible" component in the crystals of *B*-DNA. A grand canonical Monte Carlo and molecular dynamics simulation study. *J.Mol.Biol.*, **308**, 907-917.
25. Schalch, T., Duda, S., Sargent, D.F. and Richmond, T.J. (2005) X-ray structure of a tetranucleosome and its implications for the chromatin fibre. *Nature*, **436**, 138-141.
26. Song, F., Chen, P., Sun, D., Wang, M., Dong, L., Liang, D., Xu, K.-M., Zhu, P. and Li, C. (2014) Cryo-EM study of the chromatin fiber reveals a double helix twisted by tetranucleosomal units. *Science*, **344**, 376-380.
27. Baker, N.A., Sept, D., Joseph, S., Holst, M.J. and McCammon, J.A. (2001) Electrostatics of nanosystems: application to microtubules and the ribosome. *Proc. Natl. Acad. Sci. U.S.A.*, **98**, 10037-10041.
28. Pettersen, E.F., Goddard, T.D., Huang, C.C., Couch, G.S., Greenblatt, D.M., Meng, E.C. and Ferrin, T.E. (2004) UCSF Chimera - A visualization system for exploratory research and analysis. *J.Comp.Chem.*, **25**, 1605-1612.
29. Luger, K., Mader, A.W., Richmond, R.K., Sargent, D.F. and Richmond, T.J. (1997) Crystal structure of the nucleosome core particle at 2.8 Å resolution. *Nature*, **389**, 251-260.
30. Harp, J.M., Hanson, B.L., Timm, D.E. and Bunick, G.J. (2000) Asymmetries in the nucleosome core particle at 2.5 Å resolution. *Acta Cryst., sect.D*, **56**, 1513-1534.
31. Suto, R.K., Clarksson, M.J., Tremethick, D.J. and Luger, K. (2000) Crystal structure of a nucleosome core particle containing the variant histone H2A.Z. *Nature Struct.Biol.*, **7**, 1121-1124.
32. White, C.L., Suto, R.K. and Luger, K. (2001) Structure of the yeast nucleosome core particle reveals fundamental changes in internucleosome interactions. *EMBO J.*, **20**, 5207-5218.
33. Suto, R.K., Edayathumangalam, R.S., White, C.L., Melander, C., Gottesfeld, J.M., Dervan, P.B. and Luger, K. (2003) Crystal structures of nucleosome core particles in complex with minor groove DNA-binding ligands. *J.Mol.Biol.*, **326**, 371-380.
34. Muthurajan, U.M., Bao, Y., Forsberg, L.J., Edayathumangalam, R.S., Dyer, P.N., White, C.L. and Luger, K. (2004) Crystal structures of histone Sin mutant nucleosomes reveal altered protein-DNA interactions. *EMBO J.*, **23**, 260-271.

35. Edayathumangalam, R.S., Weyermann, P., Gottesfeld, J.M., Dervan, P.B. and Luger, K. (2004) Molecular recognition of the nucleosomeal "supergroove". *Proc.Natl.Acad.Sci.U.S.A.*, **101**, 6864-6869.
36. Chakravarthy, S., Gundimella, S.K., Caron, C., Perche, P.Y., Pehrson, J.R., Khochbin, S. and Luger, K. (2005) Structural characterization of the histone variant macroH2A. *Mol.Cell.Biol.* , **25**, 7616-7624.
37. Barbera, A.J., Chodaparambil, J.V., Kelley-Clarke, B., Joukov, V., Walter, J.C., Luger, K. and Kaye, K.M. (2006) The nucleosomal surface as a docking station for Kaposi's sarcoma herpesvirus LANA. *Science*, **311**, 856-861.
38. Tsunaka, Y., Kajimura, N., Tate, S. and Morikawa, K. (2005) Alteration of the nucleosomal DNA path in the crystal structure of a human nucleosome core particle. *Nucleic Acids Res.*, **33**, 3424-3434.
39. Bao, Y., White, C.L. and Luger, K. (2006) Nucleosome core particles containing a poly(dA·dT) sequence element exhibit a locally distorted DNA structure. *J.Mol.Biol.*, **361**, 617-624.
40. Ong, M.S., Richmond, T.J. and Davey, C.A. (2007) DNA stretching and extreme kinking in the nucleosome core. *J.Mol.Biol.*, **368**, 1067-1074.
41. Clapier, C.R., Chakravarthy, S., Petosa, C., Fernández-Tornero, C., Luger, K. and Müller, C.W. (2008) Structure of the Drosophila nucleosome core particle highlights evolutionary constraints on the H2A-H2B histone dimer. *Proteins*, **71**, 1–7.
42. Tachiwana, H., Kagawa, W., Osakabe, A., Kawaguchi, K., Shiga, T., Hayashi-Takanaka, Y., Kimura, H. and Kurumizaka, H. (2010) Structural basis of instability of the nucleosome containing a testis-specific histone variant, human H3T. *Proc.Natl.Acad.Sci.U.S.A.*, **107**, 10454-10459.
43. Tachiwana, H., Kagawa, W., Shiga, T., Osakabe, A., Miya, Y., Saito, K., Hayashi-Takanaka, Y., Oda, T., Sato, M., Park, S.Y. *et al.* (2011) Crystal structure of the human centromeric nucleosome containing CENP-A. *Nature*, **476**, 232-235.
44. Tachiwana, H., Osakabe, A., Shiga, T., Miya, Y., Kimura, H., Kagawa, W. and Kurumizaka, H. (2011) Structures of human nucleosomes containing major histone H3 variants. *Acta Cryst., sect.D*, **67**, 578-583.
45. Iwasaki, W., Tachiwana, H., Kawaguchi, K., Shibata, T., Kagawa, W. and Kurumizaka, H. (2011) Comprehensive structural analysis of mutant nucleosomes containing lysine to glutamine (KQ) substitutions in the H3 and H4 histone-fold domains. *Biochemistry*, **50**, 7822-7832.
46. Wu, B., Dröge, P. and Davey, C.A. (2008) Site selectivity of platinum anticancer therapeutics. *Nat.Chem.Biol.*, **4**, 110-112.
47. Lu, X., Simon, M.D., Chodaparambil, J.V., Hansen, J.C., Shokat, K.M. and Luger, K. (2008) The effect of H3K79 dimethylation and H4K20 trimethylation on nucleosome and chromatin structure. *Nat. Struct. Mol. Biol.*, **15**, 1122-1124.
48. Davey, G.E., Wu, B., Dong, Y., Surana, U. and Davey, C.A. (2010) DNA stretching in the nucleosome facilitates alkylation by an intercalating antitumour agent. *Nucleic Acids Res.*, **38**, 2081-2088.
49. Watanabe, S., Resch, M., Lilyestrom, W., Clark, N., Hansen, J.C., Peterson, C. and Luger, K. (2010) Structural characterization of H3K56Q nucleosomes and nucleosomal arrays. *Biochim.Biophys.Acta*, **1799**, 480-486.
50. Wu, B., Mohideen, K., Vasudevan, D. and Davey, C.A. (2010) Structural insight into the sequence dependence of nucleosome positioning. *Structure*, **18**, 528-536.
51. Wu, B. and Davey, C.A. (2010) Using soft X-rays for a detailed picture of divalent metal binding in the nucleosome. *J.Mol.Biol.*, **398**, 633-640.

52. Vasudevan, D., Chua, E.Y. and Davey, C.A. (2010) Crystal structures of nucleosome core particles containing the '601' strong positioning sequence. *J.Mol.Biol.*, **403**, 1-10.
53. Mohideen, K., Muhammad, R. and Davey, C.A. (2010) Perturbations in nucleosome structure from heavy metal association. *Nucleic Acids Res.*, **38**, 6301-6311.
54. Wu, B., Ong, M.S., Groessl, M., Adhireksan, Z., Hartinger, C.G., Dyson, P.J. and Davey, C.A. (2011) A ruthenium antimetastasis agent forms specific histone protein adducts in the nucleosome core. *Chemistry*, **17**, 3562-3566.
55. Makde, R.D., England, J.R., Yennawar, H.P. and Tan, S. (2010) Structure of RCC1 chromatin factor bound to the nucleosome core particle. *Nature*, **467**, 562-566.
56. Todd, R.C. and Lippard, S.J. (2010) Consequences of cisplatin binding on nucleosome structure and dynamics. *Chem. Biol.*, **17**, 1334-1343.
57. Wu, B., Davey, G.E., Nazarov, A.A., Dyson, P.J. and Davey, C.A. (2011) Specific DNA structural attributes modulate platinum anticancer drug site selection and cross-link generation. *Nucleic Acids Res.*, **39**, 8200-8212.
58. Armache, K.J., Garlick, J.D., Canzio, D., Narlikar, G.J. and Kingston, R.E. (2011) Structural basis of silencing: Sir3 BAH domain in complex with a nucleosome at 3.0 Å resolution. *Science*, **334**, 977-982.
59. Chua, E.Y., Vasudevan, D., Davey, G.E., Wu, B. and Davey, C.A. (2012) The mechanics behind DNA sequence-dependent properties of the nucleosome. *Nucleic Acids Res.*, **40**, 6338-6352.
60. Iwasaki, W., Miya, Y., Horikoshi, N., Osakabe, A., Taguchi, H., Tachiwana, H., Shibata, T., Kagawa, W. and Kurumizaka, H. (2013) Contribution of histone N-terminal tails to the structure and stability of nucleosomes. *FEBS Open Bio*, **3**, 363-369.
61. Horikoshi, N., Sato, K., Shimada, K., Arimura, Y., Osakabe, A., Tachiwana, H., Hayashi-Takanaka, Y., Iwasaki, W., Kagawa, W., Harata, M. *et al.* (2013) Structural polymorphism in the L1 loop regions of human H2A.Z.1 and H2A.Z.2. *Acta Crystallogr. D Biol. Crystallogr.*, **69(Pt 12)**, 2431-2439.
62. Urahama, T., Horikoshi, N., Osakabe, A., Tachiwana, H. and Kurumizaka, H. (2014) Structure of human nucleosome containing the testis-specific histone variant TSH2B *Acta Cryst. F*, **70**, 444–449.
63. Arimura, Y., Shirayama, K., Horikoshi, N., Fujita, R., Taguchi, H., Kagawa, W., Fukagawa, T., Almouzni, G. and Kurumizaka, H. (2014) Crystal structure and stable property of the cancer-associated heterotypic nucleosome containing CENP-A and H3.3. *Sci. Rep.*, **4**, 7115.
64. Padavattan, S., Shinagawa, T., Hasegawa, K., Kumasaka, T., Ishii, S. and Kumarevel, T. (2015) Structural and functional analyses of nucleosome complexes with mouse histone variants TH2a and TH2b, involved in reprogramming. *Biochem. Biophys. Res. Commun.*, **464**, 929-935.
65. Meier, S.M., Hanif, M., Adhireksan, Z., Pichler, V., Novak, M., Jirkovsky, E., Jakupec, M.A., Arion, V.B., Davey, C.A., K., K.B. *et al.* (2013) Novel metal(II) arene 2-pyridinecarbothioamides: a rationale to orally active organometallic anticancer agents. *Chem. Sci.*, **4**, 1837-1846.
66. Wang, F., Li, G., Altaf, M., Lu, C., Currie, M.A., Johnson, A. and Moazed, D. (2013) Heterochromatin protein Sir3 induces contacts between the amino terminus of histone H4 and nucleosomal DNA. *Proc Natl Acad Sci U S A*, **110**, 8495-8500.
67. Adhireksan, Z., Davey, G.E., Campomanes, P., Groessl, M., Clavel, C.M., Yu, H., Nazarov, A.A., Yeo, C.H., Ang, W.H., Dröge, P. *et al.* (2014) Ligand substitutions between ruthenium-cymene compounds can control protein versus DNA targeting and anticancer activity. *Nat. Commun.*, **5**, 3462.

68. Yang, D., Fang, Q., Wang, M., Ren, R., Wang, H., He, M., Sun, Y., Yang, N. and Xu, R.M. (2013) Nα-acetylated Sir3 stabilizes the conformation of a nucleosome-binding loop in the BAH domain. *Nat. Struct. Mol. Biol.*, **20**, 1116-1118.
69. Arnaudo, N., Fernández, I.S., McLaughlin, S.H., Peak-Chew, S.Y., Rhodes, D. and Martino, F. (2013) The N-terminal acetylation of Sir3 stabilizes its binding to the nucleosome core particle. *Nat. Struct. Mol. Biol.*, **20**, 1119-1121.
70. Zhou, B.R., Jiang, J., Feng, H., Ghirlando, R., Xiao, T.S. and Bai, Y. (2015) Structural mechanisms of nucleosome recognition by linker histones. *Mol. Cell*, **59**, 628-638.
71. McGinty, R.K., Henrici, R.C. and Tan, S. (2014) Crystal structure of the PRC1 ubiquitylation module bound to the nucleosome. *Nature*, **514**, 591-596.
72. Chua, E.Y., Davey, G.E., Chin, C.F., Dröge, P., Ang, W.H. and Davey, C.A. (2015) Stereochemical control of nucleosome targeting by platinum-intercalator antitumor agents. *Nucleic Acids Res.*, **43**, 5284-5296.
73. Kato, H., Jiang, J., Zhou, B.R., Rozendaal, M., Feng, H., Ghirlando, R., Xiao, T.S., Straight, A.F. and Bai, Y. (2013) A conserved mechanism for centromeric nucleosome recognition by centromere protein CENP-C. *Science*, **340**, 1110-1113.
74. Hanif, M., Meier, S.M., Adhireksan, Z., Henke, H., Martic, S., Labib, M., Kandioller, W., Jakupec, M.A., Kraatz, H.B., Davey, C.A. *et al.* Contrasting cytotoxicity and reactivity of RuII-arene complexes of morpholine vs thiomorpholine substituted 3-hydroxy-2-pyridone ligands. *To Be Published*
75. Chatterjee, N., North, J.A., Dechassa, M.L., Manohar, M., Prasad, R., Luger, K., Ottesen, J.J., Poirier, M.G. and Bartholomew, B. (2015) Histone acetylation near the nucleosome dyad axis enhances nucleosome disassembly by RSC and SWI/SNF. *Mol. Cell. Biol.*, **35**, 4083-4092.
76. Osakabe, A., Tachiwana, H., Kagawa, W., Horikoshi, N., Matsumoto, S., Hasegawa, M., Matsumoto, N., Toga, T., Yamamoto, J., Hanaoka, F. *et al.* (2015) Structural basis of pyrimidine-pyrimidone (6-4) photoproduct recognition by UV-DDB in the nucleosome. *Sci. Rep.*, **5**, 16330.
77. Urahama, T., Harada, A., Maehara, K., Horikoshi, N., Sato, K., Sato, Y., Shiraishi, K., Sugino, N., Osakabe, A., Tachiwana, H. *et al.* (2016) Histone H3.5 forms an unstable nucleosome and accumulates around transcription start sites in human testis. *Epigenetics Chromatin*, **9**, 2.
78. Morgan, M.T., Haj-Yahya, M., Ringel, A.E., Bandi, P., Brik, A. and Wolberger, C. (2016) Structural basis for histone H2B deubiquitination by the SAGA DUB module. *Science*, **351**, 725-728.
79. Wakamori, M., Fujii, Y., Suka, N., Shirouzu, M., Sakamoto, K., Umehara, T. and Yokoyama, S. (2015) Intra- and inter-nucleosomal interactions of the histone H4 tail revealed with a human nucleosome core particle with genetically-incorporated H4 tetra-acetylation. *Sci. Rep.*, **5**, 17204.
80. Kujirai, T., Horikoshi, N., Sato, K., Maehara, K., Machida, S., Osakabe, A., Kimura, H., Ohkawa, Y. and Kurumizaka, H. (2016) Structure and function of human histone H3.Y nucleosome. *Nucleic Acids Res.*, **44**, 6127-6141.
81. Suzuki, Y., Horikoshi, N., Kato, D. and Kurumizaka, H. (2016) Crystal structure of the nucleosome containing histone H3 with crotonylated lysine 122. *Biochem. Biophys. Res. Commun.*, **469**, 483-489.
82. Ueda, J., Harada, A., Urahama, T., Machida, S., Maehara, K., Hada, M., Makino, Y., Nogami, J., Horikoshi, N., Osakabe, A. *et al.* (2017) Testis-specific histone variant H3t gene is essential for entry into spermatogenesis. *Cell Rep.*, **18**, 593-600.
83. Horikoshi, N., Tachiwana, H., Kagawa, W., Osakabe, A., Matsumoto, S., Iwai, S., Sugasawa, K. and Kurumizaka, H. (2016) Crystal structure of the nucleosome

containing ultraviolet light-induced cyclobutane pyrimidine dimer. *Biochem. Biophys. Res. Commun.*, **471**, 117-122.

84. Fujii, Y., Wakamori, M., Umehara, T. and Yokoyama, S. (2016) Crystal structure of human nucleosome core particle containing enzymatically introduced CpG methylation. *FEBS Open Bio.*, **6**, 498-514.
85. Horikoshi, N., Arimura, Y., Taguchi, H. and Kurumizaka, H. (2016) Crystal structures of heterotypic nucleosomes containing histones H2A.Z and H2A. *Open Biol.*, **6**, 160127.
86. Machida, S., Sekine, S., Nishiyama, Y., Horikoshi, N. and Kurumizaka, H. (2016) Structural and biochemical analyses of monoubiquitinated human histones H2B and H4. *Open Biol.*, **6**, 160090.
87. Ma, Z., Palermo, G., Adhireksan, Z., Murray, B.S., von Erlach, T., Dyson, P.J., Rothlisberger, U. and Davey, C.A. (2016) An organometallic compound which exhibits a DNA topology-dependent one-stranded intercalation mode. *Angew. Chem. Int. Ed. Engl.*, **55**, 7441-7444.
88. Osakabe, A., Adachi, F., Arimura, Y., Maehara, K., Ohkawa, Y. and Kurumizaka, H. (2015) Influence of DNA methylation on positioning and DNA flexibility of nucleosomes with pericentric satellite DNA. *Open Biol.*, **5**, 150128.
89. Adhireksan, Z., Palermo, G., Riedel, T., Ma, Z., Muhammad, R., Rothlisberger, U., Dyson, P.J. and Davey, C.A. (2017) Allosteric cross-talk in chromatin can mediate drug-drug synergy. *Nat. Commun.*, **8**, 14860.
90. Fang, Q., Chen, P., Wang, M., Fang, J., Yang, N., Li, G. and Xu, R.M. (2016) Human cytomegalovirus IE1 protein alters the higher-order chromatin structure by targeting the acidic patch of the nucleosome. *Elife*, **5**, e11911.
91. Frouws, T.D., Duda, S.C. and Richmond, T.J. (2016) X-ray structure of the MMTV-A nucleosome core. *Proc. Natl. Acad. Sci. U. S. A.*, **113**, 1214-1219.
92. Kato, D., Osakabe, A., Arimura, Y., Mizukami, Y., Horikoshi, N., Saikusa, K., Akashi, S., Nishimura, Y., Park, S.Y., Nogami, J. *et al.* (2017) Crystal structure of the overlapping dinucleosome composed of hexasome and octasome. *Science*, **356**, 205-208.
93. Padavattan, S., Thiruselvam, V., Shinagawa, T., Hasegawa, K., Kumasaka, T., Ishii, S. and Kumarevel, T. (2017) Structural analyses of the nucleosome complexes with human testis-specific histone variants, hTh2a and hTh2b. *Biophys. Chem.*, **221**, 41-48.
94. Amamoto, Y., Aoi, Y., Nagashima, N., Suto, H., Yoshidome, D., Arimura, Y., Osakabe, A., Kato, D., Kurumizaka, H., Kawashima, S.A. *et al.* (2017) Synthetic posttranslational modifications: Chemical catalyst-driven regioselective histone acylation of native chromatin. *J. Am. Chem. Soc.*, **139**, 7568-7576.
95. Taguchi, H., Xie, Y., Horikoshi, N., Maehara, K., Harada, A., Nogami, J., Sato, K., Arimura, Y., Osakabe, A., Kujirai, T. *et al.* (2017) Crystal structure and characterization of novel human histone H3 variants, H3.6, H3.7, and H3.8. *Biochemistry*, **56**, 2184-2196.
96. Girish, T.S., McGinty, R.K. and Tan, S. (2016) Multivalent interactions by the Set8 histone methyltransferase with its nucleosome substrate. *J. Mol. Biol.*, **428**, 1531-1543.
97. Osakabe, A., Arimura, Y., Matsumoto, S., Horikoshi, N., Sugasawa, K. and Kurumizaka, H. (2017) Polymorphism of apyrimidinic DNA structures in the nucleosome. *Sci. Rep.*, **7**, 41783.
98. Wilson, M.D., Benlekbir, S., Fradet-Turcotte, A., Sherker, A., Julien, J.P., McEwan, A., Noordermeer, S.M., Sicheri, F., Rubinstein, J.L. and Durocher, D. (2016) The structural basis of modified nucleosome recognition by 53BP1. *Nature*, **536**, 100-103.

99. Lesbats, P., Serrao, E., Maskell, D.P., Pye, V.E., O'Reilly, N., Lindemann, D., Engelman, A.N. and Cherepanov, P. (2017) Structural basis for spumavirus GAG tethering to chromatin. *Proc. Natl. Acad. Sci. U.S.A.*, **114**, 5509-5514.

100. Bednar, J., Garcia-Saez, I., Boopathi, R., Cutter, A.R., Papai, G., Reymer, A., Syed, S.H., Lone, I.N., Tonchev, O., Crucifix, C. *et al.* (2017) Structure and dynamics of a 197 bp nucleosome in complex with linker histone H1. *Mol. Cell*, **66**, 384-397.e388.

101. Liu, X., Li, M., Xia, X., Li, X. and Chen, Z. (2017) Mechanism of chromatin remodelling revealed by the Snf2-nucleosome structure. *Nature*, **544**, 440-445.
